# Consensus on DEfinition of Food Allergy SEverity (DEFASE) an integrated mixed methods systematic review

**DOI:** 10.1016/j.waojou.2020.100503

**Published:** 2021-03-11

**Authors:** Stefania Arasi, Ulugbek Nurmatov, Audrey Dunn-Galvin, Shahd Daher, Graham Roberts, Paul J. Turner, Sayantani B. Shinder, Ruchi Gupta, Philippe Eigenmann, Anna Nowak-Wegrzyn, Mario A. Sánchez Borges, Ignacio J. Ansotegui, Montserrat Fernandez-Rivas, Stavros Petrou, Luciana Kase Tanno, Marta Vazquez-Ortiz, Brian P. Vickery, Gary Wing-Kin Wong, Motohiro Ebisawa, Alessandro Fiocchi

**Affiliations:** a Allergy Unit - Area of Translational Research in Pediatric Specialities, Bambino Gesù Children's Hospital, IRCCS, Rome, Italy; b Division of Population Medicine, School of Medicine, Cardiff University, Wales, UK; c Applied Psychology and Paediatrics and Child Health, University College Cork, Cork, Ireland; d Nuffield Department of Primary Care Health Sciences, University of Oxford, Radcliffe Observatory Quarter, England, UK; e NIHR Southampton Biomedical Research Centre, University Hospital Southampton NHS Foundation Trust, Clinical and Experimental Sciences and Human Development in Health, Faculty of Medicine, University of Southampton, Southampton, UK; f The David Hide Asthma and Allergy Research Centre, St Mary's Hospital, Isle of Wight, UK; g National Heart & Lung Institute, Imperial College London, London, UK; h Discipline of Paediatrics and Child Health, School of Medicine, University of Sydney, Sydney, Australia; i Division of Pulmonary, Allergy, and Critical Care Medicine, Department of Medicine, Stanford University, Sean N. Parker Center for Allergy and Asthma Research at Stanford University, Stanford University, Stanford, CA, USA; j Center for Food Allergy and Asthma Research (CFAAR), Northwestern University Feinberg School of Medicine, Department of Pediatrics & Medicine, Ann & Robert H. Lurie Children's Hospital of Chicago, USA; k Pediatric Allergy Unit, Department of Women-Children-Teenagers Pediatrics, University Hospitals of Geneva, Geneva, Switzerland; l Allergy and Immunology, Department of Pediatrics, New York University School of Medicine, Langone Health, New York, NY, USA; m Department of Pediatrics, Gastroenterology and Nutrition, Collegium Medicum, University of Warmia and Mazury, Olsztyn, Poland; n Allergy and Clinical Immunology Department, Centro Médico Docente La Trinidad, Caracas, Venezuela; o Department Allergy and Immunology, Hospital Quironsalud Bizkaia, Bilbao, Spain; p Servicio de Alergia, Hospital Clınico San Carlos, UCM, IdISSC, ARADyAL, Madrid, Spain; q Hospital Sírio Libanês, São Paulo, Brazil; r University Hospital of Montpellier, Montpellier, France; s Sorbonne Universités, Paris, France; t Department of Paediatrics, Imperial College London, United Kingdom; u Department of Pediatrics, Emory University, Atlanta, GA, USA; v Department of Paediatrics, The Chinese University of Hong Kong, Prince of Wales Hospital, Shatin, Hong Kong, China; w Clinical Research Center for Allergy and Rheumatology, National Hospital Organization, Sagamihara National Hospital, Kanagawa, Japan

**Keywords:** Definition, Food allergy, Severity, Systematic review, Mixed-methods study

## Abstract

**Background and aims:**

The term “Food Allergy” refers to a complex global health problem with a wide spectrum of severity. However, a uniform definition of severe food allergy is currently missing. This systematic review is the preliminary step towards a state-of-the-art synopsis of the current evidence relating to the severity of IgE-mediated food allergy; it will inform attempts to develop a consensus to define food allergy severity by clinicians and other stakeholders.

**Methods:**

We undertook a mixed-methods systematic review, which involved searching 11 international biomedical databases for published studies from inception to 31 December 2019. Studies were independently screened against pre-defined eligibility criteria and critically appraised by established instruments. The substantial heterogeneity of included studies precluded meta-analyses and, therefore, narrative synthesis of quantitative and qualitative data was performed.

**Results:**

We found 23 studies providing eligible primary data on symptom-specific severity of food allergic reactions, and 31 previously published symptom-severity scoring systems referred to food allergic reactions. There were seven studies which assessed quality-of-life measures in patients (and family members) with different food allergy severity and two studies that investigated the economic burden of food allergy severity. Overall, the quality and the global rating of all included studies were judged as being moderate.

**Conclusions:**

There is heterogeneity among severity scoring systems used and even outcomes considered in the context of severity of food allergy. No score has been validated. Our results will be used to inform the development of an international consensus to define the severity of food allergy.

**Systematic review registration:**

A protocol was prospectively registered with the International Prospective Register of Systematic Reviews (PROSPERO) database with the registration number CRD42020183103 (https://www.crd.york.ac.uk/prospero/#recordDetails).

## Background and rationale

Over the last few decades, with increasing prevalence, food Allergy (FA) has emerged as a global health problem affecting up to 10% of the population.^1^, ^2^, ^3^, ^4^ Epidemiological studies demonstrate an increase not only in prevalence, particularly in children,^5^^,^^6^ but also in severity with remarkable morbidity and in some cases, mortality.^7^^,^^8^ A diagnosis of FA can result in a significant adverse impact on health-related quality of life for both allergic individuals and their families, with an increase in emotional, social, and financial burdens.^9^ However, as for other diseases, including allergic pathologies, there are different phenotypes of FA with variability in allergen-specific clinical symptoms and eliciting doses.^10^ Patients with milder forms, such as those with only oral symptoms, are certainly worthy of diagnostic attention, but may not require all the therapeutic and management resources that are necessary for the patient at higher risk of life-threatening food-induced anaphylaxis. Diagnosing FA remains highly complex, and although numerous biomarkers are under exploration,^11^^,^^12^ the most commonly used tests remain food allergen-specific immunoglobulin E (sIgE) and skin prick testing (SPT) which do not correlate with severity.^13^ Presently, without a reliable classification system, we risk treating all FA patients in the same way, in effect, a one-size-fits-all approach that is unhelpful to patients, their families, and their providers. Severity classifications are available for both allergic rhinitis and asthma; however, for FA, no such specific scoring system for classifying severity currently exists.^14^^,^^15^

Without a standardized classification system in place, terminology and definitions that are currently in use are not comparable across studies and among different stakeholders.^16^ Standardizing the classification of FA severity will benefit not just patients and providers, but also patient advocacy groups, disease registries, research, food and drug industries, government agencies and regulators, as well as legislative bodies. There is presently a great need for an international consensus-based system to define FA severity.

World Allergy Organization (WAO) is undertaking the development of an international definition and classification of severity associated with food allergy (“**DE**finition of **F**ood **A**llergy **SE**verity”, DEFASE). The preliminary step in the formulation of a uniform definition and classification of FA severity includes a state-of-the-art synopsis of the current evidence. This systematic review focuses exclusively on IgE-mediated food allergy (ie, acute allergic reactions manifesting as a broad spectrum of signs/symptoms ranging from urticaria to vomiting and wheezing, up to fatal or near-fatal anaphylaxis).^17^ To our knowledge, this is the first systematic review of the literature on current severity classifications used for FA.

## Methods

This systematic review (SR) was conducted by a panel of allergy specialists, psychologists, other health-care professionals, economists, academicians, researchers, patient representatives, and methodologists. The members of DEFASE team come from Europe, North and South America, Asia, and Australasia.

### Plan of investigation

The methods are briefly described herein. A detailed SR protocol “Consensus on the DEfinition of Food Allergy SEverity (DEFASE): protocol for an integrated mixed methods systematic review” was registered with International Prospective Register of Systematic Reviews (PROSPERO) CRD42020183103 and accepted for publication in World Allergy Organization Journal.

### Population

Studies on patients of any age with a physician-confirmed diagnosis of IgE-mediated food allergies to eggs, milk, peanuts, tree nuts, and/or any other food were eligible for inclusion.

### Outcomes

Our outcomes of interest were: (a) symptom-related severity scores for food allergic reactions; (b) non-symptom related severity scores for food allergy, ie, health-related quality of life and economic evaluations; (c) methodological approaches used to derive definitions of food allergy severity; (d) the features used to define them (ie, variables, either clinical [eg, type and numbers of reactions to the culprit food; comorbidities; cofactors; disease-related quality of life impairment], or immunological characteristics [eg, pattern of sensitization to allergenic molecules, IgE-specific activities]); (e) the characteristics associated with severity category.

We considered and categorised food allergy severity as either symptom-related or non-symptom-related severity scores. We have considered all scores designed for or applied to food induced allergic reactions. However, we included primary data on symptom severity of food allergic reactions only from papers reporting on physician confirmed diagnosis of IgE mediated food allergy based on a positive history and IgE sensitization (skin prick test, SPT) and/or serum levels of specific IgE (sIgE) with/without oral food challenge (OFCs). We also evaluated validated scoring systems for food allergy quality of life (FAQL) and how FAQL and/or food allergy independent measure (FAIM) is impacted by "severity" of food allergic reactions in patients with physician - confirmed food allergy.

This mixed-methods SR was designed to capture and include all types of partial and full economic evaluations of food allergy severity. The economic evaluations could either be partial economic evaluations (cost analyses or cost-cost offset analyses) or they could be full economic evaluations that identify, measure, and value costs and outcomes of the severity of food allergy with an appropriate comparator(s). The different types of full economic evaluations include cost-effectiveness analyses, cost-utility analyses, cost-benefit analyses, cost-consequence analyses, and cost-minimisation analyses. The results were analysed to determine the number of studies that support the severity of food allergy on cost-effectiveness grounds, and where available an overall recommendation was made based on the results of partial economic evaluations (eg, cost analysis).

### Study types

Papers whose primary or secondary aim is to define or identify severity classifications of IgE-mediated food allergies in humans were considered eligible for inclusion in our SR. The following study types were eligible for inclusion:

• All analytical studies: ie, cohort, case-control, and cross-sectional studies; case series involving 40 or more participants; and economic evaluation of FA severity.
• All interventional studies: ie, randomized controlled trials, RCT; quasi- RCTs; controlled clinical trials, CCT; interrupted time series, ITS; and controlled before after studies.

In addition, we also included reviews, SRs, guidelines, position and consensus papers, editorials, and rostrums.

The following study types were excluded: studies of non-IgE mediated food allergy; studies that used only self-reported diagnosis of food allergy; primary data from studies on allergen immunotherapy; non-research letters and editorials; case reports; case-series with less than 40 participants; and in-progress phenotyping studies (abstracts) as they are unlikely to provide sufficient detail on the definitions of food allergy severity score; animal studies; and studies that examined food allergy as a predictor of a separate outcome (eg, asthma development).

### Research methods for identification of studies: electronic databases

We systematically searched 11 international databases: AMED (1985–2019); CAB (1910–2019); CINAHL (1937–2019); Cochrane Library (1992–2019); Econlit (1886–2019); EMBASE (1980–2019); Global Health (1987–2019); Google Scholar (2000–2019); ISI Web of Science (which contains the Science Citation Index) (1970–2019); MEDLINE (1966–2019); TRIP (2003–2019).

### Search strategy for electronic databases

A search strategy was developed in Medline format and adopted for other databases. MEDLINE and EMBASE databases were searched using the controlled vocabulary search terms (MeSH and EMTREE, respectively) combined using Boolean terminology with a wide-range of free-text terms. The results were limited to humans (see Online Supplementary material, section “Search strategy”). There were no publication year or publication status restrictions; however, the searches were restricted to only English language. Searches were undertaken from inception up to 31 December 2019.

### Additional search methods

All references of published studies were hand searched. The bibliographies of all eligible studies were scrutinised to identify possible additional studies. In addition, we contacted the primary study authors to clarify discordant data [Table S1]. We also reviewed the reference lists of relevant studies.

### Study selection

Duplicate publications were removed. Titles and abstracts of identified studies were checked against the inclusion/exclusion criteria independently by two reviewers.

Full-text papers were retrieved if their titles and/or abstracts appeared to meet the eligibility criteria or if the decision could not be made based on the titles and/or abstracts alone. Assessment of the full texts of each retrieved paper was undertaken independently by two reviewers using the same criteria. Disagreements about inclusion were resolved through discussion at the meetings.

### Assessment of methodological quality

The methodological quality of included observational studies was independently assessed by two reviewers (UN, SA) by using the Effective Public Health Practice Project (EPHPP).^18^ We focused on the following domains to assess the quality of included studies: selection bias; study design; confounders; blinding; data collection method; withdrawals and dropouts; and final global rating. Each component-specific parameter (ie, suitability of the study design for the research question; risk of selection bias; exposure measurement; outcome assessment; and generalizability of findings) was given a judgment: “strong”; “moderate”; and “weak”. At the end of critical appraisal, we also provided the overall grading for each study.

### Data extraction

Data were independently extracted onto a customized data extraction sheet by two reviewers (SA, UN), and any discrepancies were resolved by discussion or, if agreement was not reached, the third reviewer arbitrated.

### Meta-analysis

Meta-analysis was inappropriate given the substantial heterogeneity of the populations, exposures, outcomes and study designs.

## Results

### An overview: characteristics of included studies

**Fig. 1:**
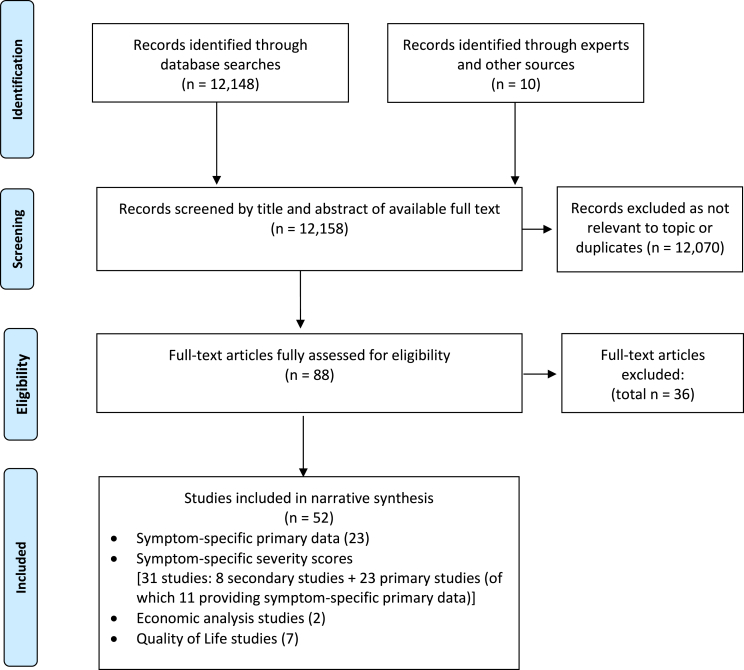
PRISMA flow diagram

Our searches identified 12 148 potentially relevant papers and 10 further papers identified by experts; 2365 duplicate papers were removed; a further 9705 papers were excluded for not meeting our inclusion criteria. Furthermore, 88 papers were at full text level, and in total 52 studies satisfied our inclusion criteria and were thus included in our systematic review (see Fig. 1, PRISMA flow diagram). Manuscripts excluded at full-text screening phase and reasons for exclusion are explained in Table S2.

We found 23 studies providing eligible primary data on symptom-specific severity of food allergic reactions^4^^,^^19^, ^20^, ^21^, ^22^, ^23^, ^24^, ^25^, ^26^, ^27^, ^28^, ^29^, ^30^, ^31^, ^32^, ^33^, ^34^, ^35^, ^36^, ^37^, ^38^, ^39^, ^40^ [Table S3].

Fourteen studies reported aggregated symptom-specific primary data referred to allergic reactions triggered by any allergenic source (ie, not only by food allergens). We tried to contact the respective authors several times; however, only in three studies, primary data referred specifically to food allergic reactions were provided by the contacted authors [Table S1] and, therefore, those three studies have been included^28^^,^^34^^,^^35^ in the category of primary data.

In terms of study design, the 23 eligible studies were: 12 cohort;^19^^,^^21^^,^^27^^,^^29^^,^^33^, ^34^, ^35^, ^36^, ^37^, ^38^, ^39^, ^40^ two case-control;^4^^,^^20^ four cross-sectional;^22^^,^^23^^,^^30^^,^^41^ and five case series studies.^24^, ^25^, ^26^^,^^28^^,^^32^

Additionally, our SR identified 31 previously published symptom-severity scoring systems referred to food allergic reactions^1^^,^^4^^,^^15^^,^^19^^,^^20^^,^^22^^,^^24^^,^^26^^,^^27^^,^^29^, ^30^, ^31^, ^32^, ^33^^,^^42^, ^43^, ^44^, ^45^, ^46^, ^47^, ^48^, ^49^, ^50^, ^51^, ^52^, ^53^, ^54^, ^55^, ^56^, ^57^, ^58^ [Table 1]. Twenty-three were primary studies providing new symptom-severity scoring systems to assess food-induced allergic reactions^1^^,^^4^^,^^19^^,^^20^^,^^22^^,^^24^^,^^26^^,^^27^^,^^29^, ^30^, ^31^, ^32^, ^33^^,^^42^, ^43^, ^44^, ^45^, ^46^, ^47^, ^48^, ^49^, ^50^^,^^58^ [Table 1, Table 2]. Of note, we were able to pool primary data eligible for our SR only from 11 of them.^4^^,^^19^^,^^20^^,^^22^^,^^24^^,^^26^^,^^27^^,^^29^, ^30^, ^31^, ^32^ The remaining 20 studies provided new symptom-severity scores but not eligible primary data for our SR since they: a) included allergic reactions triggered by a different^58^ or any allergenic source (ie, not only food);^46^, ^47^, ^48^^,^^50^ b) or were based on self-reported diagnosis of food allergy;^1^^,^^42^^,^^45^^,^^49^ c) or included food allergy diagnosis only based on IgE-sensitization without history of ingestion of the suspected culprit food;^44^ d) or referred to oral immunotherapy trials.^43^ Eight out of the 31 that included symptom-severity scores were provided by secondary research papers.^15^^,^^51^, ^52^, ^53^, ^54^, ^55^, ^56^, ^57^ The following four secondary studies were from international collaboration, specifically, from: World Health Organization (WHO);^51^ European Academy of Allergy and Clinical Immunology (EAACI),^15^^,^^52^ and PRACTALL;^56^ two studies from Germany,^53^^,^^54^ two studies were from the United States,^55^ and one study from Sweden.^57^

**Table 1:**
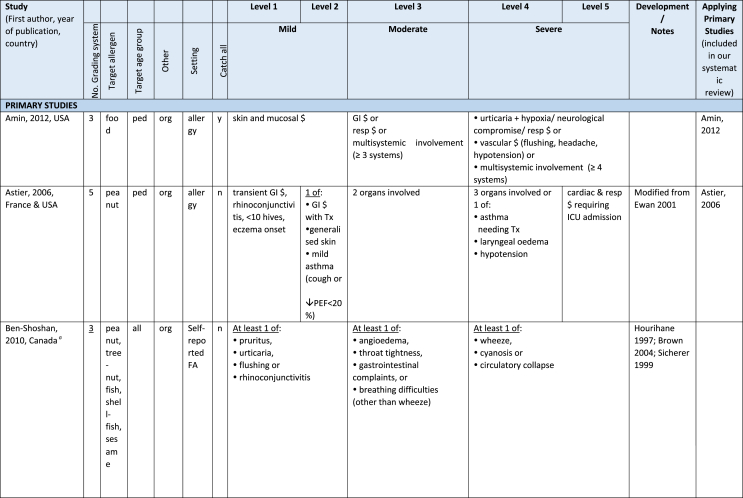

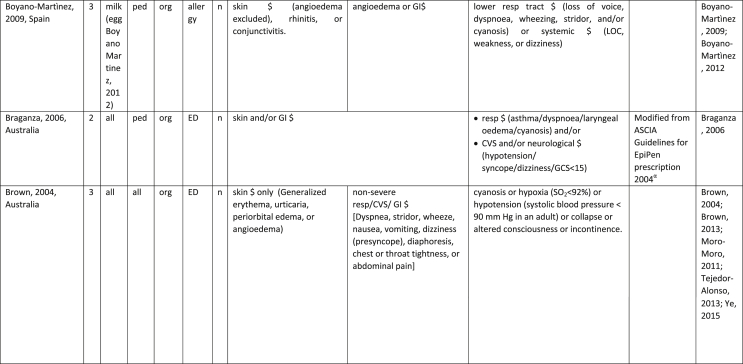

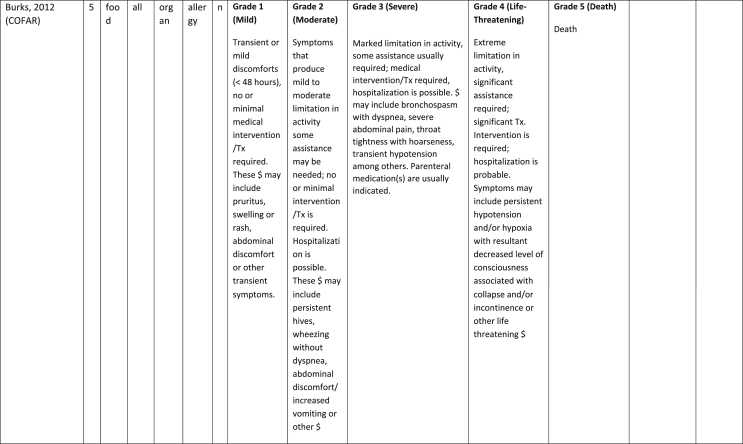

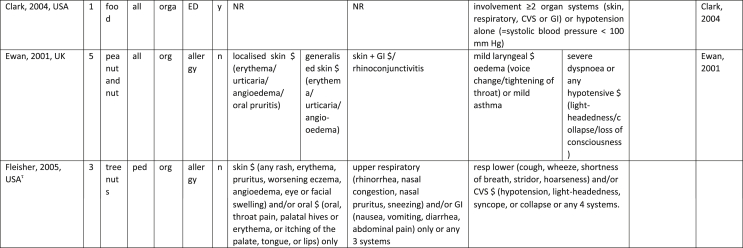

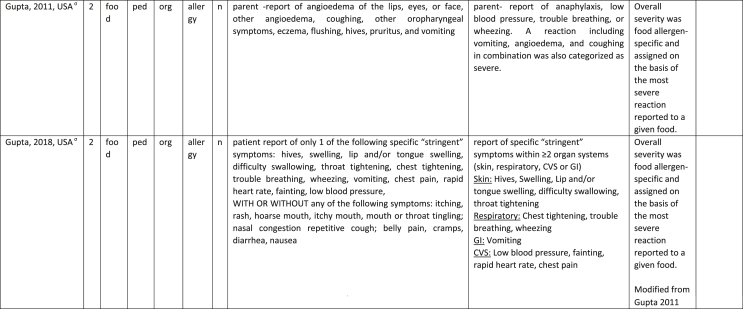

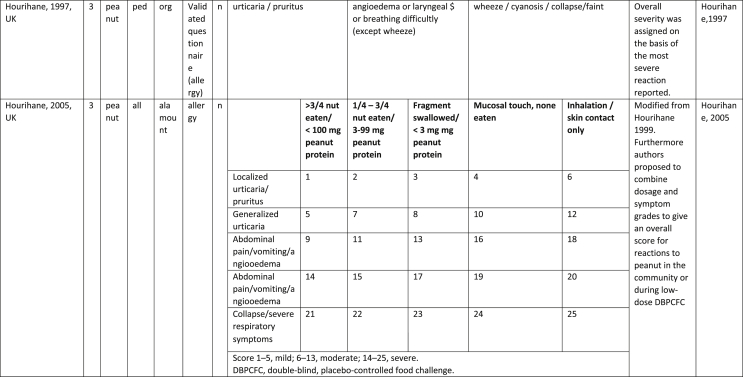

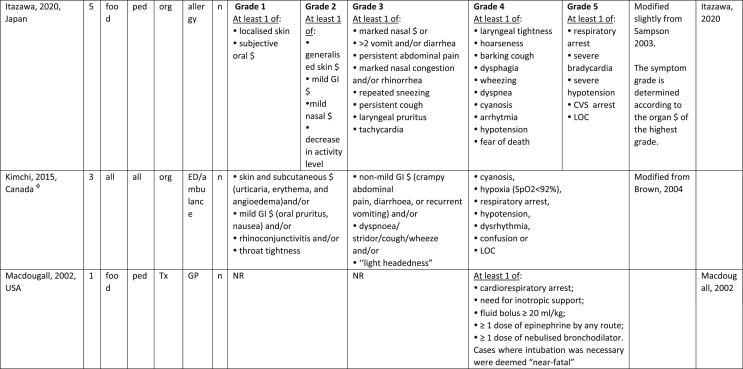

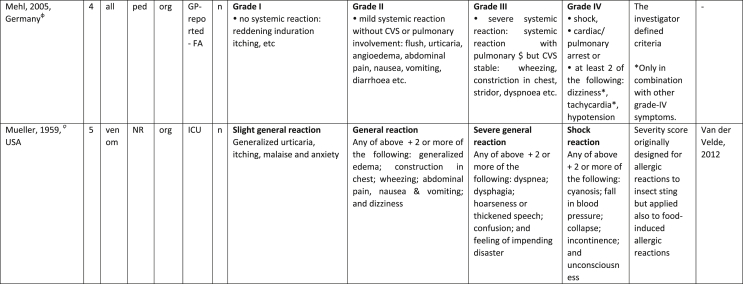

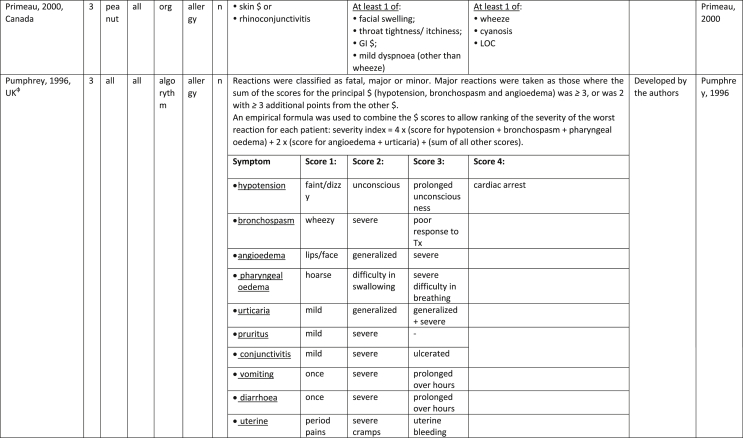

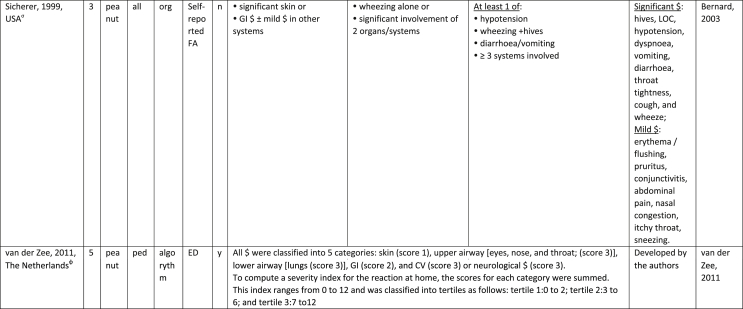

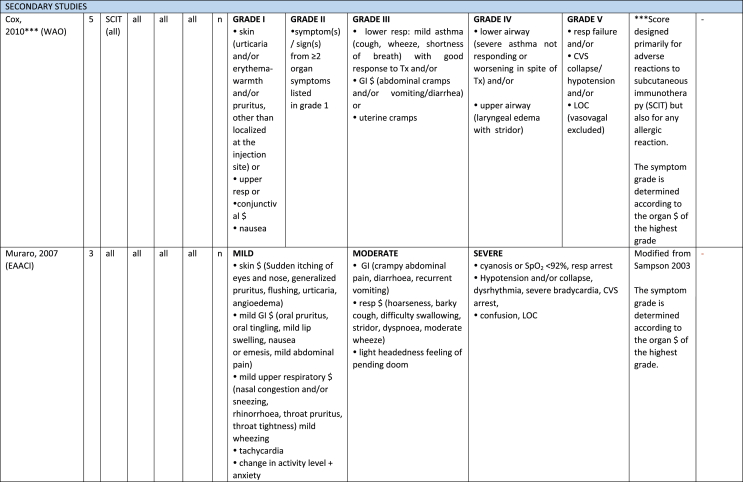

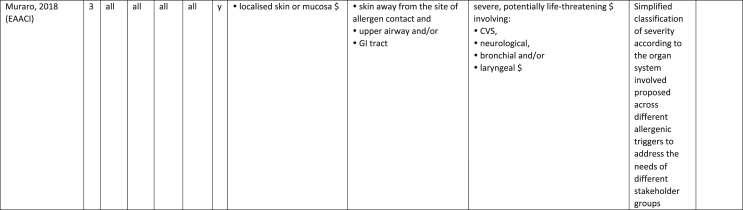

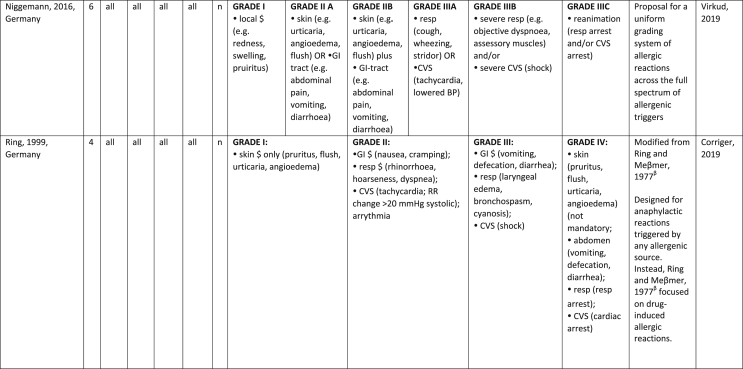

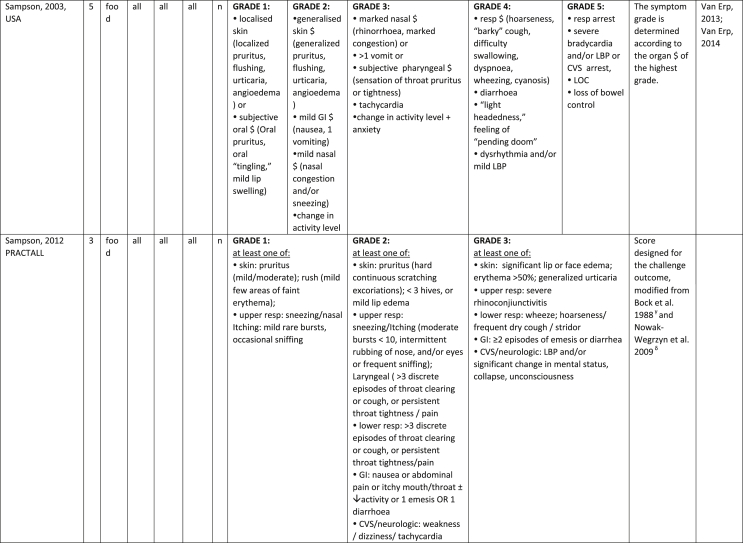

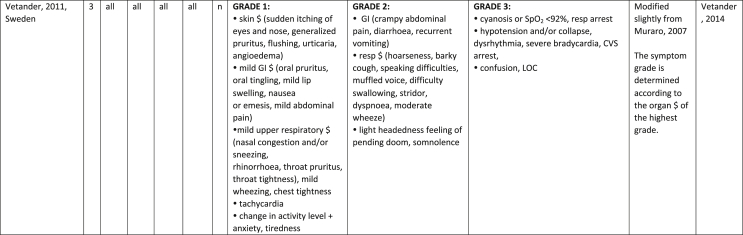
Symptom-severity scoring systems for food allergy in included primary and secondary studies

**Table 2:**
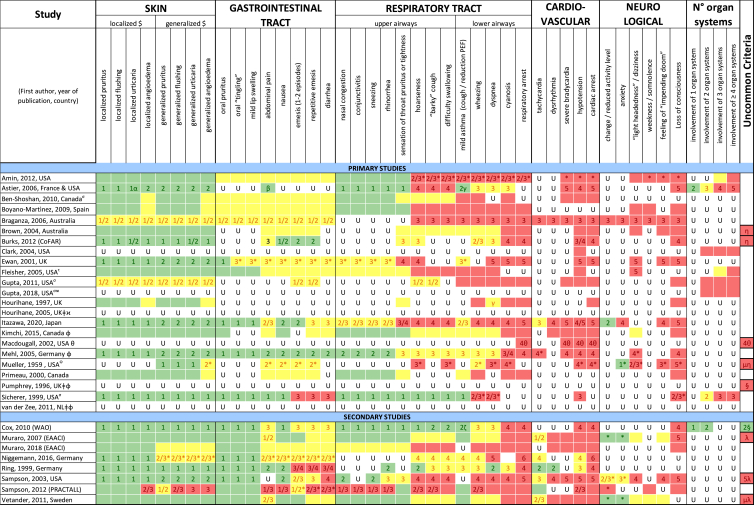

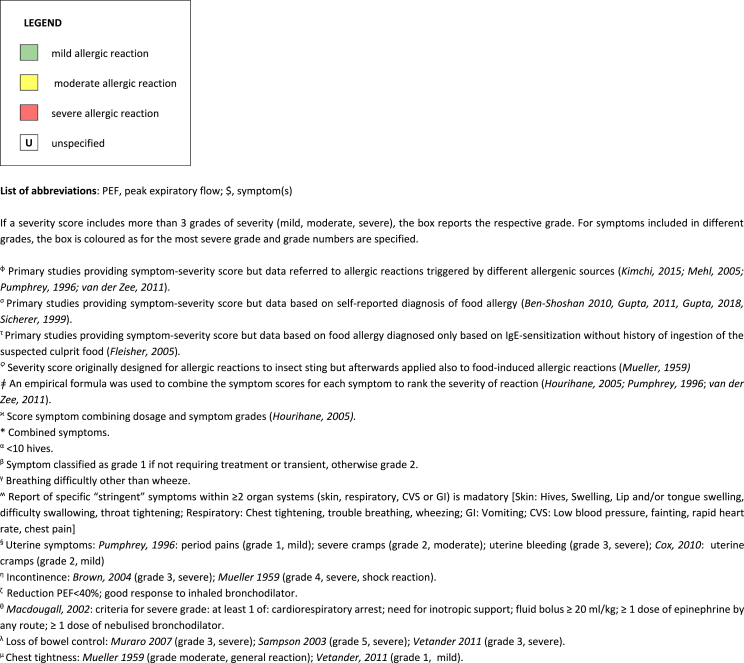
Overview of all included symptom-severity scores in included studies for each listed symptom ordered by organ

There were seven studies which assessed quality of life (QoL) measures in patients (and family members) with different food allergy severity.^59^, ^60^, ^61^, ^62^, ^63^, ^64^, ^65^ All were primary studies, with five out of the seven employing a cross-sectional,^59^^,^^61^, ^62^, ^63^^,^^65^ and two a longitudinal,^60^^,^^64^ design [Table S4].

Two studies investigated the economic burden of food allergy severity^66^^,^^67^ [Table S5].

The studies were undertaken in Australia (n = 3); Canada (n = 4); France (n = 1); Germany (n = 3); Japan (n = 1); Korea (n = 1); Spain (n = 4); Sweden (n = 2); The Netherlands (n = 4); United Kingdom (n = 5); United States (n = 14); and international collaboration (n = 10).

### Critical appraisal of studies

Quality assessment of the 23 included primary studies on symptom-severity assessment suggested that out of 12 cohort studies 10 were judged as strong.^19^^,^^29^^,^^34^, ^35^, ^36^, ^37^, ^38^, ^39^, ^40^^,^^68^ Out of two case-control studies one was judged as moderate and two as weak. Three cross-sectional studies^22^^,^^23^^,^^30^ were judged as moderate, and one study^31^ was judged as weak. Among the five case-series, two were judged as moderate,^24^^,^^26^ and three studies were judged as weak^25^^,^^28^^,^^32^ [Table S6A].

Each of the seven included studies on QoL utilized a cross-sectional design. In terms of critical appraisal, four of them have been judged as moderate^60^^,^^61^^,^^64^^,^^65^ and three as weak^59^^,^^62^^,^^63^ [Table S6B].

The absence of full economic evaluation studies in this SR precluded the use of Consolidated Health Economic Evaluation Reporting Standards (CHEERS) checklist for critical appraisal of only two cost-evaluation studies.

### Primary data on symptom-specific food allergy severity

#### Food allergy diagnosis

We included primary data on symptom severity of food allergic reactions only from papers reporting on physician confirmed diagnosis of IgE mediated food allergy based on a positive history and IgE sensitization (SPT and/or serum levels of specific IgE) with/without OFCs. In 10 out of 23 included primary studies assessing symptom severity, food allergy was confirmed by OFC.^20^, ^21^, ^22^, ^23^^,^^30^, ^31^, ^32^^,^^36^, ^37^, ^38^ Among these 10 studies, 1^31^ used a validated questionnaire with a sensitivity of 100% and a specificity of 87% for detecting peanut allergy compared with the gold standard of double-blind placebo-controlled food challenge (DBPCFC).^41^

#### Setting

The assessment of food allergy severity was carried out in different emergency departments (EDs) of hospitals in nine studies.^24^, ^25^, ^26^^,^^28^^,^^34^^,^^35^^,^^39^^,^^40^^,^^68^ Food allergy severity was assessed by allergist specialist consultations in 15 studies.^4^^,^^19^, ^20^, ^21^, ^22^, ^23^^,^^29^, ^30^, ^31^, ^32^, ^33^^,^^35^, ^36^, ^37^, ^38^

#### Recurrence of adverse reactions

Recurrence of adverse reactions (ARs) were reported in 10 studies.^22^^,^^23^^,^^28^, ^29^, ^30^, ^31^^,^^35^^,^^36^^,^^38^^,^^40^ However, these studies reported data in different formats, and we could not pool data statistically.

Epinephrine use was reported in 11 studies;^22^^,^^27^, ^28^, ^29^, ^30^, ^31^, ^32^^,^^35^^,^^36^^,^^38^^,^^40^ ED admission was recorded in five studies;^22^^,^^23^^,^^27^^,^^31^^,^^40^ and admission to hospital was provided in two studies.^28^^,^^30^ Admission to intensive care unit (ICU) was recorded in six studies,^22^, ^23^, ^24^^,^^28^^,^^31^^,^^38^ ranging from 0 up to 1.1%.

### Symptom-severity scoring systems for food allergy

Our SR identified 31 previously published instruments focusing on severity of food allergic reactions^1^^,^^4^^,^^15^^,^^19^^,^^20^^,^^22^^,^^24^^,^^26^^,^^27^^,^^29^, ^30^, ^31^, ^32^, ^33^^,^^42^, ^43^, ^44^, ^45^, ^46^, ^47^, ^48^, ^49^, ^50^, ^51^, ^52^, ^53^, ^54^, ^55^, ^56^, ^57^, ^58^ [Table 1].

Twenty-three were primary studies providing new symptom-severity scoring systems to assess food-induced allergic reactions^1^^,^^4^^,^^19^^,^^20^^,^^22^^,^^24^^,^^26^^,^^27^^,^^29^, ^30^, ^31^, ^32^, ^33^^,^^42^, ^43^, ^44^, ^45^, ^46^, ^47^, ^48^, ^49^, ^50^^,^^58^ [Table 1, Table 2]. We included eight additional symptom-severity scores from secondary literature (eg, editorials, rostrum, consensus reports, theoretical reviews, position papers).^15^^,^^51^, ^52^, ^53^, ^54^, ^55^, ^56^, ^57^

#### Setting

The instruments have been designed and developed in several settings [Table 1]: allergy specialist centres (including clinical trials)^4^^,^^19^^,^^20^^,^^22^^,^^29^^,^^30^^,^^32^^,^^43^^,^^44^^,^^48^^,^^50^ or emergency rooms^24^^,^^26^^,^^27^ or ambulance^46^ or intensive care unit^58^ or general practitioner setting^33^^,^^47^ or self (/parental) reported survey.^1^^,^^42^^,^^45^^,^^49^ Three instruments have been designed for the context of OFC.^30^^,^^32^^,^^56^

#### Targeted age group

Thirteen instruments have been originally created targeting pediatric allergic patients. All of them were primary studies.^1^^,^^19^^,^^20^^,^^22^^,^^24^^,^^31^, ^32^, ^33^^,^^43^, ^44^, ^45^^,^^47^^,^^50^ One old primary study did not report age of participants.^58^ The other 17 scores, including all of those reported in secondary literature, are applicable to all age groups^4^^,^^15^^,^^26^^,^^27^^,^^29^^,^^30^^,^^42^^,^^43^^,^^46^^,^^48^^,^^49^^,^^51^, ^52^, ^53^, ^54^, ^55^, ^56^, ^57^ [Table 1].

#### Targeted allergenic source

We included 17 symptom-severity scoring systems primarily designed to assess allergic reactions elicited by food^1^^,^^4^^,^^19^^,^^20^^,^^22^^,^^29^, ^30^, ^31^, ^32^, ^33^^,^^42^, ^43^, ^44^, ^45^^,^^49^^,^^50^^,^^68^ [Table 1] as follows: seven for any food;^1^^,^^19^^,^^27^^,^^32^^,^^33^^,^^43^^,^^45^ six for peanut only;^4^^,^^20^^,^^30^^,^^31^^,^^49^^,^^50^ one for peanut and nuts;^29^ 1 for peanut, tree-nuts, fish, shell-fish, and sesame;^42^; one for tree-nuts;^44^ one for milk^22^ but also applied to egg.^23^ One score has been created to assess severity of food induced reactions occurring during oral immunotherapy.^43^ We identified 11 instruments created to evaluate the severity of allergic reactions triggered by any allergenic source, including food.^15^^,^^24^^,^^26^^,^^46^, ^47^, ^48^^,^^51^, ^52^, ^53^, ^54^^,^^57^ One of them was designed by authors primarily for adverse reactions to subcutaneous immunotherapy (SCIT) but with the indication to be applied for any allergic reaction.^51^ In particular, secondary literature, highlighted the need to identify a uniform instrument to be applied to any allergenic sources at any patient age by different stakeholders.^15^^,^^51^, ^52^, ^53^, ^54^^,^^57^ Our SR included also one symptom scoring system that was originally designed to assess the overall severity of an allergic reaction elicited from other allergic condition (ie, hymenoptera venom allergy) but that has been applied afterwards to classify food-induced allergic reactions.^58^

#### Organ-specific symptoms

All included scoring systems had organ-specific outcomes covering the whole spectrum of clinical symptoms and signs in the context of IgE-mediated allergic reactions. Several of them used the term “anaphylaxis” to describe the entire spectrum of severity, although non-anaphylactic milder symptoms neither fulfil the main current definitions of anaphylaxis^69^, ^70^, ^71^, ^72^, ^73^ nor the new ICD-11 criteria.^74^

Of note, all scoring systems divided symptoms according to their anatomical involvement, ie, skin, respiratory, gastro-intestinal (GI), cardio-vascular (CVS), or neurological subjective symptoms/objective signs. Table 2 provides a detailed overview of the 30 included symptom-severity scores for each listed symptom ordered by organ. At least two reading levels of Table 2 are possible. A macroscopic evaluation suggests that there is overall concordance in assigning a progressive severity grade proceeding from the left (overall coloured in green) to the right side (in red colour), ie, spanning from skin involvement up to the lower respiratory tract, cardio- and neurological involvement passing by the gastrointestinal and upper respiratory tract (in yellow). However, a closer evaluation highlights the presence of some heterogeneities.

The majority of scoring systems used a detailed predefined list of symptoms, each of them presented as a dichotomous variable (ie, “present/non present”) or, in some cases, as a detailed grading of specific symptoms, (eg, urticaria, into mild/local or severe/generalized). A few used a more general "catch-all symptoms" approach for specific organ/system to embrace all possible symptoms for that specific organ/system (eg, all symptoms related to the “GI tract”).^15^^,^^19^^,^^27^^,^^50^ All scoring systems utilized an ordinal scale ranging over 2–6 incomparable steps. The majority of them defined the overall allergic reaction severity based on the organ symptom(s) of the highest grade (ie, most severe symptoms).^4^^,^^15^^,^^31^^,^^32^^,^^51^, ^52^, ^53^^,^^55^ Some considered the number of organ-systems involved^1^^,^^19^^,^^20^^,^^27^^,^^44^^,^^45^^,^^49^^,^^51^ or the fulfilment of “2-or-more” symptoms/organs.^58^ Three scoring tools used a mathematical formula/summation of symptoms to obtain symptom severity regardless of number of observed symptoms.^30^^,^^48^^,^^50^ Two studies considered explicitly the need of medical treatment as a criterion for assessing the symptom-severity.^33^^,^^43^ One scoring system correlated the severity of any allergic symptom to the amount of exposed food allergen.^30^

### Studies investigating predictors for symptom-severity of food allergy

This SR assessed if the included studies identified any predictors for symptom-severity of food allergy. We found that 13 included primary studies reported on the assessment of host-related and food allergen-related factors, including demographic, clinical and/or laboratory variable(s), as predictors for severe allergic reaction to food^19^, ^20^, ^21^, ^22^, ^23^^,^^26^^,^^29^, ^30^, ^31^^,^^33^^,^^36^^,^^38^^,^^39^ [Table S2].

#### Host-related factors

Three studies reported on the assessment of gender as a predictor for severity of allergic reactions; all of them reported no significant results.^23^^,^^30^^,^^36^

Six papers evaluated age as a potential parameter associated with increased risk of symptom-severity;^26^^,^^29^, ^30^, ^31^^,^^36^^,^^38^ only two studies found that adolescence and adulthood are risk factors.^29^^,^^31^

Asthma has been analized and reported as a predictor for severe ARs by five studies describing this analysis,^22^^,^^23^^,^^30^^,^^31^^,^^33^ one^31^ reported that patients with a clinical history of asthma were more likely to suffer severe ARs (x^2^ = 17.9, P.00013) and, of note, wheeze as the most common severe symptom of AR (~40% of pts). In another study, the frequency of severe ARs compared with moderate, mild, or no ARs was 10-fold higher in asthmatic children but did not reach statistical significance (OR, 10.19; 95% CI, 1.13–91.54; P .022).^22^

One study evaluated the concomitant use of drugs ie, ACE inhibitor and β-blocker) but no correlation with symptom severity was found.^26^

Three studies evaluated the role of recurrence of ARs as a predictor. The symptom-severity of the previous AR(s)^33^ or the first AR^23^ did not significantly predict the symptom-severity of the next, in the two studies reporting on this outcome.^23^^,^^33^ Similarly, one study evaluating if a previous reaction to peanut in the clinical history predicted symptom severity in peanut allergic patients found no correlation.^36^

#### Food allergen-related predictors

Two of the included studies assessed the type of food as potential risk factor.^19^^,^^39^ One found that wheat was the only predictor of severe anaphylaxis (OR 2.425, 95% CI 1.054–5.581, p < 0.037).^39^ The second study found the highest (but statistically non-significant) risk of severe ARs for peanuts (OR = 1.76, 95%CI: 0.9–3.45) and shellfish (OR = 1.54, 95%CI: 0.49–5.64) and the lowest for sesame, soy, and wheat.^19^

One study reported total IgE level as a protective factor.^23^ Total IgE levels were significantly lower in patients with moderate/severe ARs (adjusted odds ratio for every 1-unit increase in the decimal logarithm, 0.16; 95% CI, 0.05–0.54; P = .001).

On the other side, five out of the eight studies^20^, ^21^, ^22^, ^23^^,^^30^^,^^31^^,^^36^^,^^38^ reporting on serum level of specific IgE (sIgE) as a predictor for symptom severity found it as a risk factor.^21^, ^22^, ^23^^,^^30^^,^^38^ The remaining three studies found no significant results for sIgE as predictor.^20^^,^^31^^,^^36^ Of note, the five studies assessed specific IgE level to different culprit foods: sIgE to cow's milk and to casein;^22^ sIgE to egg white;^23^ and sIgE to whole peanut proteins,^21^^,^^30^ and rAra h 1, rAra h 2.^21^ In particular, one study reported that sIgE to peanut and challenge score correlated significantly in the whole group but this correlation was stronger in adults than in children, despite the median values of peanut sIgE being similar; in adults Spearman's r-value increased to 0.766 (P = 0.001, compared with children (r = 0.49, P = .018).^30^

Another study reported that age, sIgE and SPT to almond at challenge when combined demonstrated good predictive value for grade 2/3 allergic reactions by AUC (area under the curve, 0.83).^38^

A further study found that patients monosensitized to rAra h 2 had a significantly lower severity score than those polysensitized to the same source (i.e. rAra h 2 and rAra h 1 and/or rAra h 3) (P < .02).^20^ Two studies reported on SPT itself with no significant results.^20^^,^^31^

### Quality of life studies

Our SR identified 7 papers that met our inclusion criteria, namely that the studies used a validated scoring system to measure FAQL, and reported how this scoring is impacted by "severity" (symptoms/anaphylaxis). All papers included participants with confirmed FA by specialist/allergist [Table S4]. We note here that the majority of recent papers not included in the review investigated the impact of Allergy Immunotherapy on FAQL.

#### Setting and population samples

The majority of studies recruited participants through allergy specialty clinics,^59^, ^60^, ^61^^,^^64^ and two studies also recruited through general medical clinics, community support groups and media advertisements.^62^^,^^63^^,^^65^ All studies took place in The Netherlands, Ireland, and United States. The measures used were distributed through hospital allergy clinics either on site or online through the clinic to patients diagnosed with food allergy (or parents of patients diagnosed with food allergy).

#### Measures

All studies used a validated age appropriate version of the Food Allergy Quality of Life Questionnaires (FAQLQ) which are recommended as gold standard by EAACI. The FAQLQ includes questions on demographics, symptoms, reaction history, diagnosis, prescription. and use of an auto-injector. The FAQLQ also incorporates the Food Allergy Independent Measure (FAIM) which assesses the perception of severity/chance of adverse outcome, if an allergen is accidentally ingested. FAIM also operates as an anchor instrument for the FAQLQ. The instruments used were designed for data collection in general and treatment settings, cross-sectionally and longitudinally, and have reported a minimal clinical important difference (MCID) score of 0.45/0.5.

The version of the FAQLQ chosen reflected the population(s) targeted in the study. The Parent Form was used in two studies;^59^^,^^60^ the Child Form (CF) and Teen Form (TF) were used in three studies^59^^,^^61^^,^^64^ and the Adult Form in three studies.^61^^,^^62^^,^^64^ One study used only the FAIM section of FAQLQ,^63^ with all other studies using the FAIM in addition to the FAQLQ, and 1^65^ used the Parental Burden (PB) version of FAQLQ.

In addition to FAQLQ, generic measures were used to measure outcomes in three studies, namely Parental Empowerment Scale,^65^ CHQ-CF87 and Rand-36,^61^ Food Insecurity Scale (FIS), and use of food assistance programs (SNAP, food banks).^63^

#### Severity

At minimum to satisfy the inclusion criteria, all studies included questions on reaction history (eg, a list of symptoms reflecting all levels of severity), diagnosis (eg, how and by whom a patient had been diagnosed) and whether an epinephrine auto-injector (EAI) had been prescribed and reported how FAQL is impacted by "severity" (eg, symptoms/reactions). Severe food allergy was defined as having a prescription for an EAI, or self-reported previous episodes of anaphylaxis (ie, the symptoms “difficulty breathing”, “inability to stand”, collapse and/or loss of consciousness).

#### Outcomes targeted

The majority of the studies were carried out for psychometric purposes, specifically to assess the longitudinal validity and responsiveness of the FAQLQ-AF, FAQLQ-TF, FAQLQ-CF^64^ and the FAQLQ-PF^60^ and cross-cultural validity^60^^,^^62^ of the adult form (AF) and parent form (PF) respectively, and one study compared FAQL measured with generic and disease-specific questionnaires.^61^ The impact of a food challenge on FAQL was evaluated in three studies.^59^^,^^60^^,^^64^ Relationships between allergen severity, type, or comorbidities and FAQL was the focus of 2 in the context of parental empowerment,^65^ and uncertainty or inability to meet family food requirements (FIS).^63^

#### Findings/results

FAQLQ was found to be responsive to change in a food-allergic patient population with disease-specific clinical outcomes^60^^,^^64^ with good cross-cultural validity.^60^^,^^62^ All studies identified positive associations between FAQLQ impact on was found according to severity, positive challenge result, number of allergens avoided, and number of symptoms. The FAQL of American food-allergic adults was found to be more impaired than Dutch food-allergic adults^62^ and Irish food-allergic children.^60^ Caregivers classified as FIS reported an increased perceived risk of accidental ingestion, severe reaction, and death, and it was also associated with utilization of food assistance programs and food banks.^63^ Mothers reported greater empowerment and worse FAQL compared with fathers, regardless of allergen severity, type, or comorbidities, but was not significantly associated with FAQL for mothers or fathers. Highest FAQLQ-PF impact was for items involving fear of allergen exposure outside the home.^65^

### Economic burden

A SR was conducted to identify and summarise evidence regarding economic analyses of food allergy severity. Of the final articles selected for full screening, two met inclusion criteria.^66^^,^^67^ Articles that did not identify grades of food allergy severity (mild, moderate and severe) were excluded.

The first study^66^ was based on 402 cases of severe anaphylaxis reported by the Allergy Vigilance Network, in years 2004, 2005, and 2006. The setting was hospital and general practices in France. International classification of Diseases codes for anaphylaxis used in the study, included T780 (shock due to adverse food reaction), T782 (anaphylactic shock, not specified), T805 (shock due to serum/vaccine/immunization), T886 (shock due to adverse drug reactions), and T882 (anaesthetic shock). Direct and indirect costs were estimated from a national perspective. Direct costs consisted of the costs of medications, consultations, use of emergency units, diagnosis, and hospitalisations, as well as nonmedical costs such as transport, and diet. Indirect costs were based on the costs of absenteeism with a mean of three days (two days at the time of event, and one day after an event). Indirect cost data was calculated on the basis of Belgian costs.^66^

#### Direct medical costs

Results indicated that the average direct cost was €1580 per patient, ranging from €74.88 to €4445.47 (as the currency year was not indicated, it was assumed to be one year prior to the year of the publication). Costs were equivalent in purchasing power to €1,889 per patient, with a range of €89.51 to €5,314.08, in year 2020. Table S8 includes the direct medical costs for severe anaphylaxis management obtained from Flabbee et al, 2008, and adjusted to the currency year of 2020. The hospitalization had the greatest cost, ranging from €239.08 for Emergency ambulance brigade called, to severe cases of hospitalization in intensive care unit with an average cost of €2,528.25/day.

#### Indirect costs

Indirect costs were estimated to be €315 per patient; equivalent to €376.55 in year 2020.

#### Total costs

The total average cost per patient was €1895, equivalent to the cost of €2,265.27 in 2020.

The second study,^67^ investigated health service costs for food allergic individuals in Europe (Greece, Iceland, Poland, Spain, Czech Republic, France, Italy, The Netherlands, and United Kingdom), and the relationship between severity and the cost of illness. The time frame was from January 2007 until July 2009. The Geary-Khamis dollar (I$) was used to estimate unit costs of services at 2016 prices. The setting was in general practitioners’ patient lists, city council registration databases, local authority/hospital debases, and primary schools. Participants were recruited through the EuroPrevall study in a case-control study design, and completed an economic questionnaire. Participants with possible food allergy were identified by clinical history, and those with sIgE were defined as having probable allergy.^67^ Results indicated that the average health care cost for adults with possible food allergy was I$2016 (equivalent to €1 933.61 in year 2020) and I$1089 (€1 044.49 in year 2020) for controls. For children aged 7–11 years, the costs are I$2197 (€2 107.2 in year 2020) for those with possible food allergy and for controls it was I$863 (€827.73 in year 2020). The mean average yearly cost of possible and probable food allergy was I$1778 (€1705.33 in currency year 2020) in 9 participating centres. The study indicated that the cost of treating individuals with moderate allergy symptoms was 68% higher than for those with mild symptoms. Health care costs for those with severe food allergy were estimated to be double the amount for those with mild food allergy. No significant differences in health care costs were observed for children when compared with adults.

## Discussion

### Summary of main findings

To our best knowledge, this mixed methods SR represents the most comprehensive investigation ever undertaken of literature on current classification of food allergy severity.

We have tried to cover all the perspectives of food allergy severity from different stakeholders, including patients and parents/families, patient advocacy groups, disease registries, health professionals, researchers, academicians, food and drug industries, government agencies and regulators, as well as legislative bodies, as they all perceive the concept of severity differently. All included studies were observational studies that investigated symptom-specific and non-symptom specific severity of food allergy in children and adults.

We found that many severity scoring systems for food allergic reactions have been generated; however, they are heterogeneous and none of them has been validated in practice. They differ for number of steps and are only partially interchangeable. No standardized or validated method exists to compare multiple heterogeneous scoring systems. The inconsistency and non-validity of these scoring systems highlights the urgent need to develop a harmonised, consensus-based definition for the severity of food allergy in children and adults useful for all stakeholders involved.

The severity spectrum of an allergic reaction can range from subjective local mild symptoms to fatal anaphylactic shock. Type of allergen, dosage, individual threshold, route of exposure, age, comorbidity, and involvement of cofactors may influence the severity of a food allergic reaction. In turn, these variables make severity difficult to capture. Furthermore, onset and severity of each symptom can lead to progression and interaction of symptoms. Allergic reactions can occur in different incomparable settings: they range from accidental exposure in an unknown environment to controlled titrated oral food challenges in a highly specialized clinical setting.

The variability between instruments included in this SR was overall wide: some instruments are purposed solely for single allergens (eg, peanut), others developed exclusively for specific populations (ie, children) and some to specific situations (eg, OFC).

At present, the global severity of a food allergic reaction is generally either based on the highest/most severe reported symptoms or calculated by different algorithms. Some instruments used “catch-all” definitions in contrast to others based on a predefined “symptom list”, which contains more information for research purposes, and avoids the pitfall of overlooking milder symptoms.

Research into FAQL has helped to raise awareness of patient issues and provided a means of individualized assessment for a patient or parent.^75^ If a validated consensus-driven severity scoring system for FA could be developed, it could be used to harmonize outcome assessment in clinical trials and also facilitate understanding of important determinants of FAQL. This could widen the parameters of benefits and harms of new treatments and allow for the development and improvement in process and outcome quality indicators. A standardised protocol that incorporates FAQL, severity, and agreed definitions of outcomes would allow for the comparison of efficacy of food allergy treatments between centres, trials or countries. The use of such measures can help to differentiate between levels of severity on multi-dimensional patient outcomes. To this end, it is vital that improved FAQL is seen as a primary outcome, and is measured at multiple intervals during trial duration and beyond. In addition, few studies have used the minimal clinical important difference (MCID) when assessing change in FAQL. The MCID can help determine the effect of a given therapy on a patient and add meaning to statistical inferences made in research; therefore, their use is critical for the conduct and interpretability of clinical trials.

Decision science modelling is another method that could help us understand variations in preferences for treatment, which could affect the health and economic impact of allergen immunotherapy (AIT). Assessment of patient/caregiver attribute preference and how this translates to health economic outcomes will provide a basis to understand if strategies used in food allergy can deliver value-based care, which can be applied to the development of future food allergy research. Utility valuations should be derived from responses to FAQLQ instruments, providing more accurate measurement of this construct.

### Strengths and limitations of this work

To our knowledge, this is the first SR of the literature on current severity classifications used for FA. The strengths of this SR are the comprehensiveness of the searches, including all available sources of 11 international electronic databases without any geographical restrictions and with higher methodological rigour. In addition, we were able to contact an international panel of experts. We carefully grouped and categorised food allergy severity as either symptom-related or non-symptom related under several categories to find the effect of each compounds on the severity of allergic reactions to culprit food.

The methods used to verify symptom specific and non-symptom specific measures of food allergy severity were carefully assessed and graded for methodological rigour.

The main limitations of this systematic review stem from the substantial heterogeneity of studies with moderate methodological quality, to the fact that the included studies were only observational (cohort, case-control, or cross-sectional) with no interventional studies.

## Conclusions

The overall body of epidemiological evidence in relation to the food allergy severity classification is moderate. We found only observational studies, data are generally of moderate quality. This systematic review confirms the variability and diversity of severity scoring systems for FA. Overall, in terms of symptom severity systems there is a general suggestion that cardiovascular and lower respiratory tract reactions are severe and the cutaneous, and gastrointestinal ones are mild to moderate. Quality of life and economic evaluation of severity of food allergy should be incorporated into the food allergy severity definition alongside the symptom score assessment. Unfortunately, at present there is no validated and broadly accepted categorization of severity of food allergy that can be used by all stakeholders (patients, family members, guardians, healthcare professionals, researchers, food industry, policy makers and other public health authorities). A validated severity scoring system for FA could be used both for standardised patient monitoring and also to define the eligibility of allocating patients for clinical studies. This review also will represent a preliminary work for generating a consensus-based definition of severity of food allergy in children and adults, developing and implementing the algorithm by a multidisciplinary panel of experts.

There is an excess of severity scoring systems for allergic reactions including to food in children and adults. The usability of these severity scoring systems remains unclear because of methodological shortcomings, incomplete presentation, lack of internal and external validation, and testing for reliability and validity of the severity scoring systems in a range of settings and populations. The standardised, harmonised, and consensus-based uniform definition of severity scoring systems that will be used by all stakeholders is urgently needed. Rather than developing yet another severity scoring system for allergic reactions, future research should focus on external validation of scoring systems, tailoring of these models to different allergens, populations, and settings. In addition, as a gold standard, a standardised, harmonised, consensus-based severity scoring system for food allergy needs to be tested for reliability and validity in a range of settings and populations. To reach this crucial goal, expert consultation, e-Delphi study and impact studies will be the main platforms in the risk assessment and risk management of patients with food allergy [Table 3].

**Table 3:** Summary of the DEFASE systematic review

• A consensus on definition of food allergy severity is missing. This systematic review is the preliminary step towards a state-of-the-art synopsis of the current evidence relating to the severity of IgE-mediated food allergy. It will inform attempts to develop a consensus to define food allergy severity by clinicians and other stakeholders. Each participating stakeholders perspectives on food allergy severity has been covered. • All included studies were observational studies that investigated symptom-specific and non-symptom specific severity of food allergy in children and adults. • The overall body of epidemiological evidence in relation to the food allergy severity classification is moderate. • There is heterogeneity among severity scoring systems used and even outcomes considered in the context of severity of food allergy. No score has been validated. • To assess comprehensively predictors of severity of food allergy is urgently required in order to develop and use worldwide the best prediction model for severe food allergy. • Research into FAQL has helped to raise awareness of patient issues and provided a means of individualized assessment for a patient or parent. • There is lack of full economic evaluation studies on the severity of food allergy. • Shared decision making is needed to address all issues regarding the definition of food allergy severity from each stakeholder's perspectives.

## Importance to stakeholders and implementation

The concept of food allergy severity is important not only for healthcare professionals in evaluating patients, but also for patients’ family members, food and drug industries, research (clinical trials, epidemiologic, genetic, immunological, and mechanistic studies), government agencies and regulators, as well as for policy makers. The terminology and definitions currently applied to food allergy severity are not standardized, and often misleading. Furthermore, different stakeholders perceive the concept of severity differently. Therefore, a common approach is needed for an international consensus-based system to define food allergy severity and our mixed-methods systematic review comprehensively assesses and addresses this crucial issue in the management of food allergy.

## Future research

**Fig. 2:**
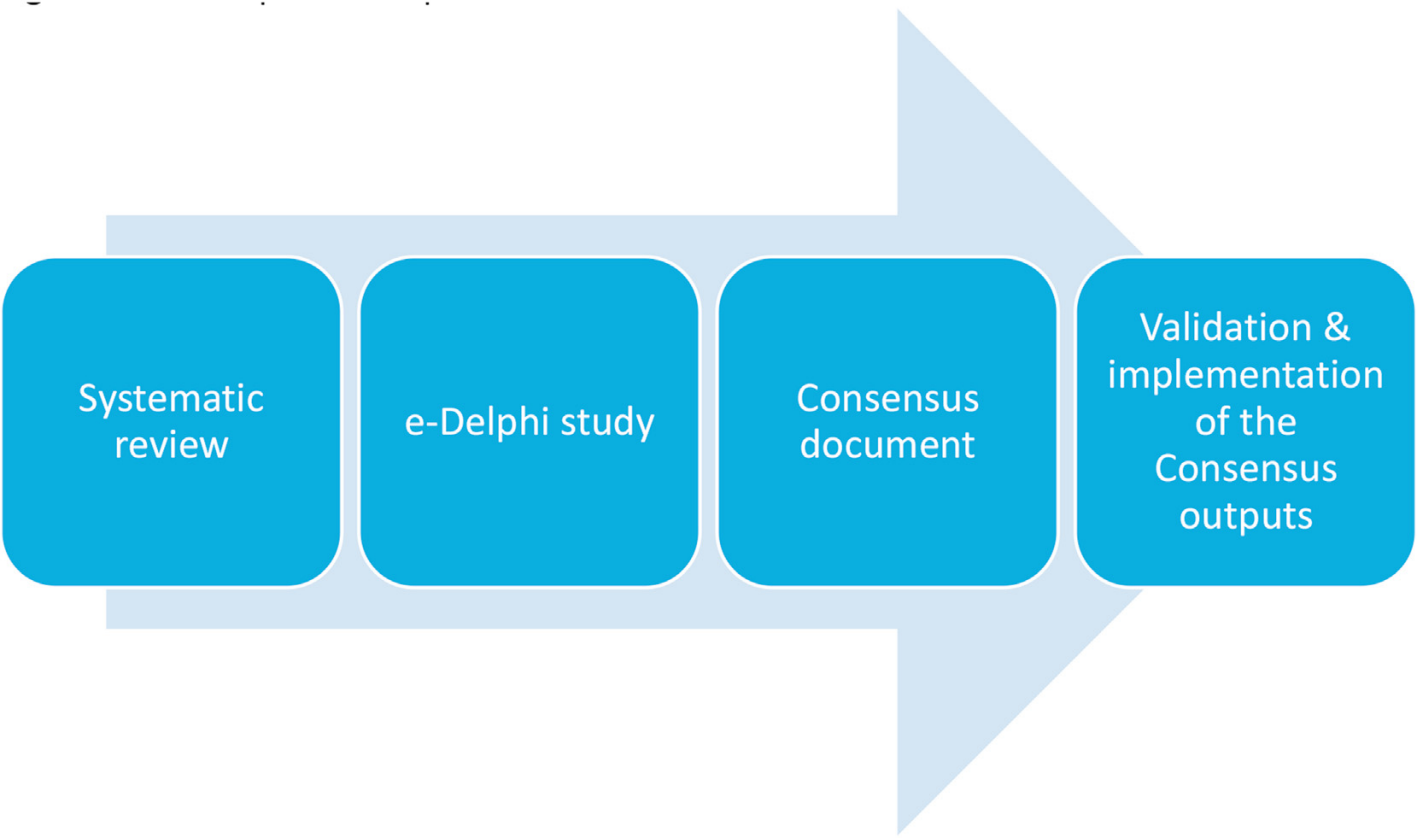
Roadmap to develop and reach the DEFASE international consensus

This SR will be a background work to reach an international consensus on the definition of food allergy severity in children and adults. As described in the roadmap (see Fig. 2), the next step will be to conduct expert consultation, through an e-Delphi study and by taking into consideration the perspectives from the different stakeholders involved. After developing the consensus document, there is a need for well-designed clinical impact studies by using large clinical databases that test the reliability and validity of severity scoring systems for food allergy. These clinical impact studies may then need to be followed up via well conducted large RCTs to evaluate the correct usage of consensus-based definitions of food allergy severity, effectiveness and cost-effectiveness of interventions aiming to reduce food allergy severity burden and risk of developing its complications in the future.

## Abbreviations

ACE, Angiotensin-Converting Enzyme; AIT, Allergen Immunotherapy; AMED, Allied and Alternative Medicine; AR, Allergic Reaction; AUC, Area Under Curve; CAB, the Commonwealth Agricultural Bureaux; CF, Child Form; CINAHL, Cumulative Index to Nursing and Allied Health Literature; CASP, Critical Appraisal Skills Programme; CBA, controlled before after studies; CCT, controlled clinical trials; CI, confidential interval; CoFAR, Consortium of Food Allergy Research; CVS, Cardio-vascular System; DBPCFC, Double-blind placebo-controlled food challenge; DEFASE, DEfinition of Food Allergy Severity; EAACI, European Academy of Allergy and Clinical Immunology; EAI, epinephrine auto-injector; EconLit, Economics Literature; ED, Emergency Department; EMBASE, Excerpta Medica database; EMTREE, EMBASE Subject Headings; EPHPP, the Effective Public Health Practice Project; FA, IgE-mediated food allergy; FAQL, food allergy quality of life; FAQLQ-AF, Food allergy quality of life questionnaire-adult form; FAQLQ-CF, Food allergy quality of life questionnaire-child form; FAQLQ-PF, Food allergy quality of life questionnaire-parent form; FAQLQ-TF, Food allergy quality of life questionnaire-teen form; FAIM, food allergy independent measure; FARE, Food Allergy Research & Education; FIS, Food Insecurity Scale; GI, Gastro-intestinal; GRADE, Grading of Recommendations Assessment, Development and Evaluation; HRQL. Health related quality of life; ICU, Intensive Care Unit; iFAAM, Integrated Approaches to Food Allergen and Allergy Risk Management; ISI, the Institute for Scientific Information; ITS, interrupted time series; MCID, Minimal clinical important difference; MEDLINE, Medical Literature Analysis and Retrieval System Online; MeSH, Medical SubHeadings; OFC, Oral Food Challenge; OR, Odds Ratio; PF, Parent Form; PRACTALL, PRACTical ALLergy; PRISMA, Preferred Reporting Items for Systematic Reviews and Meta-Analyses; PROSPERO, Prospective Register of Systematic Reviews; QoL, Quality of Life; RCT, randomized controlled studies; SCIT, Subcutaneous Immunotherapy; SNAP, the Supplemental Nutrition Assistance Program; SPT, Skin Prick Test; SR, systematic review; TF, Teen Form; TRIP, Turning Research Into Practice; USA, the United States of America; WAO, World Allergy Organization

## Funding

This is a project of the World Allergy Organization (WAO), supported by a grant from Novartis, as well as Abbott Laboratories and Food Allergy Research & Education (FARE). WAO funded the systematic review to support the development of the international consensus on the definition of severe food allergy. The funder and supporters had no role in the development of the protocol nor in the conduct of the systematic review or its publication.

## Availability of data and materials

N/A.

## Contributorship

This manuscript was drafted by SA, UN, ADG, SD. The document was reviewed by all co-authors. All authors read, provided feedback and approved the final manuscript.

## Ethics approval

Ethical approval is not required for this study as it is a systematic review. However, author's potential conflicts of interest is disclosed from the beginning.

## Consent for publication

N/A.

## Declaration of competing interest

The following authors declared no potential interests: Ignacio J Ansotegui; Stefania Arasi; Shahd Daher; Alessandro Fiocchi; Ulugbek Nurmatov; Stavros Petrou; Graham Roberts; Mario Sánchez-Borges; Luciana Kase Tanno; Marta Vazquez Ortiz; Gary Wing-Kin Wong.

Some of the authors have professional affiliations related to the content of the systematic review as set out below:

Audrey Dunn-Galvin: consultancy fee from Aimmune and DBV;

Paul J Turner: grants from UK 10.13039/501100000265Medical Research Council, 10.13039/501100013342NIHR/Imperial BRC, JM Charitable Foundation and UK 10.13039/501100000354Food Standards Agency; personal fees from UK Food Standards Agency, DBV Technologies, ILSI Europe and Allergenis, non-financial support from Aimmune Therapeutics;

Sayantani B Sindher: supported by 10.13039/100000002NIH grants. Involved in clinical trials with Regeneron, Aimmune Therapeutics, DBV Technologies, Adare Pharmaceuticals, Sanofi, Novartis;

Ruchi Gupta: grants from The 10.13039/100000002National Institute of Health (NIH) (R21 ID # AI135705, R01 ID# AI130348, U01 ID # AI138907), Rho Inc., Stanford Sean N. Parker Center for Allergy Research, UnitedHealth Group, Thermo Fisher Scientific, Genentech, and the National Confectioners Association (NCA); is employed by Ann & Robert H. Lurie Children's Hospital of Chicago; is a Professor of Pediatrics at Northwestern University; and serves as a medical consultant/advisor for Before Brands, Kaléo Inc., Genentech, ICER, FARE, Aimmune Therapeutics, and DBV Technologies;

Philippe Eigenmann: Research grants and support: Ulrich Muller Gierock Foundation, 10.13039/100011033ThermoFisher Scientific; Consultant: DBV technologies, Nesté, Danone, Novartis, Abbott; Speaking engagements: ThermoFisher Scientific, ALK, Abbott; Stock options: DBV technologies;

Anna Nowak-Wegrzyn: Grant/Research/Clinical Trial Support: DBV Technologies; ITN/NIAID; 10.13039/100004324Astellas Pharmaceutical; 10.13039/100004339Sanofi-Aventis [Future therapies for food allergy]. International FPIES Association [FPIES, unpaid advisor]. Consultant/Advisory Boards: Gerber Nutrition Institute; Merck. Speaking engagements: Nestle, Thermofisher Scientific;

Montserrat Fernandez-Rivas: research grants from the Spanish Government (10.13039/501100003329MINECO, ISCIII) for the project SOLMILK, ARADyAL network and PI19/01095; consultancy fees from Aimmune, DBV, Novartis, SPRIM; lecture fees from ALK, Allergy Therapeutics, Diater, GSK, HAL Allergy, Thermofisher Scientific;

Brian P Vickery: Employment: Pediatric Institute of Emory University + Children's Healthcare of Atlant; Consultant/Advisor: Aimmune Therapeutics; AllerGenis, LLC; Food Allergy Research and Education (FARE); Reacta Biosciences; Grant support: NIH-NIAID; 10.13039/100006423FARE; 10.13039/100004328Genentech; Clinical investigator: Aimmune; DBV Technologies; Regeneron;

Motohiro Ebisawa: lecture fees: DBV technologies and Mylan.
