# Supplementary material for: Consensus on DEfinition of Food Allergy SEverity (DEFASE) an integrated mixed methods systematic review
Source: World Allergy Organ J. 2021 Mar 11;14(3):100503. doi: 10.1016/j.waojou.2020.100503 (PMC7966874; doi:10.1016/j.waojou.2020.100503)
Supplement: Multimedia component 1 [file mmc1.pdf]

- **Supplementary Methods: Search strategy**

Search strategy_MEDLINE format

1 exp Food Hypersensitivity/

2 exp Milk Hypersensitivity/

3 exp Egg Hypersensitivity/

4 exp Peanut Hypersensitivity/

5 exp Tree nut Hypersensitivity/

6 exp Nut Hypersensitivity/

7 ((food or Oral Allergy Syndrome or milk or egg or peanut or arachis hypogaea or tree nut or hazelnut or brazil nut or walnut or chestnut or pistachio or almond or legumes or wheat or rice or soy or fish or seafood or shellfish or shrimp or lobster or crab or crawfish or kiwi or apple or peach or apricot or cherry or pear or plum or tomato or green pea or potato or carrot or parsley or celery or additives) adj3 (allerg* or hypersensitivit*)).mp.

8 Allergic reaction to food.mp.

9 exp Food allergy/

10 exp Anaphylaxis/ or severe food allergy.mp.

11 Systemic anaphylaxis/ or exp anaphylaxis/ or skin anaphylaxis/ or anaphylaxis.mp. or passive skin anaphylaxis/

12 Serious food allergy.mp.

13 allergic reaction*.mp.

14 Fatal allergic reaction*.mp.

15 (Near-fatal allergic reaction* or near fatal allergic reaction*).mp.

16 (Anaphylaxis and (adrenaline or epinephrine)).mp.

17 or/1-16

18 (Definition* or code or classif* or ICD).mp.

19 17 and 18

20 Animals/ not Humans/

21 19 not 20

22 (advertisements or animation or architectural drawings or bibliography or biography or book illustrations or bookplates or charts or comment or letter or editorial or news or patient education handout or published erratum or retraction of publication).mp. [mp=title, abstract, original title, name of substance word, subject heading word, floating sub-heading word, keyword heading word, organism supplementary concept word, protocol supplementary concept word, rare disease supplementary concept word, unique identifier, synonyms]

23 21 not 22

- **Supplementary Tables**

Table S1. The List of experts contacted

No	Author	Title	Contact person/ emails
1	*Asai et al.*	Asai Y, Yanishevsky Y, Clarke A, et al. Rate, Triggers, Severity and Management of Anaphylaxis in Adults Treated in a Canadian Emergency Department. Int Arch Allergy Immunol 2014;164:246–252	Dr. Moshe Ben-Shoshan moshebenshoshan@gmail.com
2	*Choi et al.*	Choi B, Kim SH, Lee H. Are Registration of Disease Codes for Adult Anaphylaxis Accurate in the Emergency Department? Allergy Asthma Immunol Res. 2018;10(2):137-143	Dr Sun Hyu Kim stachy1@paran.com
3	*Corriger et al.*	Corriger J, Beaudouin E, Rothmann, Penven E, Haumonte Q, Thomas H, et al. Epidemiological Data on Anaphylaxis in French Emergency Departments. J Investig Allergol Clin Immunol 2019;29(5): 357-364.	Dr. Jeremy Corriger jeremy.corriger@hotmail.fr Dr. Luciana Kase Tanno luciana.tanno@gmail.com
4	*Huang et al.*	Huang F, Chawla K, Järvinen KM, NowakWeęgrzyn A. Anaphylaxis in a New York City pediatric emergency department: triggers, treatments, and outcomes. J Allergy Clin Immunol. 2012;129(1): 162–168.e3.	Prof Anna Nowak-Węgrzyn anna.nowak-wegrzyn@mssm.edu
5	*Kimchi et al.*	Kimchi N, Clarke A, Moisan J, et al. Anaphylaxis cases presenting to primary care paramedics in Quebec. Immunity, Inflammation and Disease 2015; 3(4): 406–410	Dr. Moshe Ben-Shoshan moshebenshoshan@gmail.com
6	*Mehl et al.*	Mehl A, Wahn U, Niggemann B. Anaphylactic reactions in children – a questionnaire-based survey in Germany. Allergy 2005: 60: 1440–1445	Prof Bodo Niggemann bodo.niggemann@charite.de
7	*Moro-Moro et al.*	Moro-Moro M, Alonso MAT, Hernández JE, Garcia MVM, Ingelmo AR, Albelda CV. Incidence of Anaphylaxis and Subtypes of Anaphylaxis in a General Hospital Emergency Department. J Investig Allergol Clin Immunol 2011;21(2):142-149.	Dr. Miguel A Tejedor Alonso m914674227@telefonica.net matejedor@fhalcorcon.es
8	*Nieto-Nieto et al.*	Nieto-Nieto A, Tejedor-Alonso MA, Farias-Aquino E, Moro-Moro M,Rosado Ingelmo A, Gonzalez-Moreno A. Clinical profile of patients with severe anaphylaxis hospitalized in	Dr. Miguel A Tejedor Alonso m914674227@telefonica.net matejedor@fhalcorcon.es

		the Spanish hospital system: 1997-2011. J Investig Allergol Clin Immunol 2017; 27(2):111-126.	
9	*Oropeza et al.*	Oropeza AR, Lassen A, Halken S, Bindslev-Jensen C, Mortz CG. Anaphylaxis in an emergency care setting: a one year prospective study in children and adults. Emergency Medicine 2017:25:111	Dr. Ruiz Oropeza<br>athamaica.ruiz.oropeza@rsyd.dk
10	*Piromrat et al.*	Piromrat K, Chinratanapisit S, Trathong S. Anaphylaxis in an emergency department: a 2-year study in a tertiary-care hospital. Asian Pacific Journal of Allergy and Immunology 2008;26:121-128.	Dr. Kanika Piromrat<br>drkanika2004@yahoo.com
11	*Santaella et al.*	Santaella ML, Cox PR, Ramos C, Dosdier OM. Anaphylaxis: an analysis of cases evaluated at the Puerto Rico Medical Center over a ten-year period. Anaphylaxis in Puerto Rico PRHSJ 2006;25(2):143-147.	Dr. Maria L Santaella<br>lousant@prtc.net
12	*Tanno L et al.*	Tanno L, Molinari N, Bruelet S, et al. Field-testing the new anaphylaxis' classification for the WHO International Classification of Diseases-11 revision. Allergy 2017; 72: 820–826.	Dr Luciana Tanno<br>luciana.tanno@gmail.com
13	*Tejedor - Alonso et al.*	Alonso MA, Garcia MV, Hernandez JE, Moro MM, Ezquerra PE, Ingelmo AR, Albelda CV. Recurrence of anaphylaxis in a Spanish series. Journal of investigational allergology & clinical immunology. 2013;23(6):383-391.	Dr. Miguel A Tejedor-Alonso<br>m914674227@telefonica.net<br>matejedor@fhalcorcon.es
14	*Topal et al.*	Topal E, Bakirtas A, Yilmaz O, et al. Epidemiological and Clinical Features of Anaphylaxis: Single Center Experience with 109 Children. Pediatric Allergy, Immunology and Pulmonology 2013;26(2):88-92.	Dr Erdem Topal<br>erdemtopal44@gmail.com

Table S2. Manuscripts excluded at full-text screening phase and reasons for exclusion

First author	year of publication	reference	REASON FOR EXCLUSION
**Asai**	2014	Asai Y, Yanishevsky Y, Clarke A, et al. Rate, triggers, severity and management of anaphylaxis in adults treated in a Canadian emergency department. Int Arch Allergy Immunol. 2014;164(3):246-52.	data referred to allergic reactions due to different allergenic triggers. Data on food-induced allergic reactions can not be extrapolated and have not been provided by authors after requests. Previously reported grading system.
**Barbi**	2012	Barbi E, Longo G, Berti I, et al. Adverse effects during specific oral tolerance induction: in-hospital "rush" phase. Eur Ann Allergy Clin Immunol. 2012;44(1):18-25.	food allergy oral immunotherapy
**Branum**	2012	Branum AM, Simon AE, Lukacs SL. Among children with food allergy, do sociodemographic factors and healthcare use differ by severity?. Matern Child Health J. 2012;16 Suppl 1(0 1):S44-S50.	self-reported (parental report), not physician-diagnosed food allergy
**Brown**	2001	Brown AF, McKinnon D, Chu K. Emergency department anaphylaxis: A review of 142 patients in a single year. J Allergy Clin Immunol. 2001;108(5):861-6.	data referred to allergic reactions due to different allergenic triggers. Data on food-induced allergic reactions can not be extrapolated and have not been provided by authors after requests. Previously reported grading system.
**Burks**	2012	Burks AW, Jones SM, Wood RA, Fleischer DM, Sicherer SH, Lindblad RW, et al. Oral immunotherapy for treatment of egg allergy in children. N Engl J Med. 2012;367(3):233-43	food allergy oral immunotherapy
**Castells**	2017	Castells M. Diagnosis and management of anaphylaxis in precision medicine. J Allergy Clin Immunol. 2017;140(2):321-333.	severity score referred to drug allergy

**Choi**	2018	Choi B, Kim SH, Lee H. Are Registration of Disease Codes for Adult Anaphylaxis<br>Accurate in the Emergency Department? Allergy Asthma Immunol Res. 2018;10(2):137-143.	data referred to allergic reactions due to different allergenic triggers. Data on food-induced allergic reactions can not be extrapolated and have not been provided by authors after requests. Previously reported grading system.
**Choi**	2019	Choi B, Kim SH, Lee H. Missed Registration of Disease Codes for Pediatric Anaphylaxis at the Emergency Department. Emerg Med Int. 2019 Aug 14;2019:4198630. doi: 10.1155/2019/4198630.	data referred to allergic reactions due to different allergenic triggers. Data on food-induced allergic reactions can not be extrapolated and have not been provided by authors after requests. Previously reported grading system.
**Cianferoni**	2001	Cianferoni A, Novembre E, Mugnaini L, et al. Clinical features of acute anaphylaxis in patients admitted to a university hospital: an 11-year retrospective review (1985-1996). Ann Allergy Asthma Immunol. 2001;87(1):27-32.	no severity score reported
**Collignon**	2011	Collignon, Monnez JM, Vallois P, et al. Discriminant analyses of peanut allergy severity scores, Journal of Applied Statistics. 2011;38:9, 1783-1799.	wrong study design [discriminant analyses of DBPCFC score to formulate predictive models]
**De Schryver**	2019	De Schryver S, Mazer B, Clarke AE, et al. Adverse Events in Oral Immunotherapy for the Desensitization of Cow's Milk Allergy in Children: A Randomized Controlled Trial. J Allergy Clin Immunol Pract. 2019;7(6):1912-1919	food allergy oral immunotherapy
**Dibek Misirlioglu**	2017	Dibek Misirlioglu E, Vezir E, Toyran M, Capanoglu M, Guvenir H, Civelek E, Kocabas CN. Clinical diagnosis and management of anaphylaxis in infancy. Allergy Asthma Proc. 2017;38(1):38-43.	data referred to allergic reactions due to different allergenic triggers. Data on food-induced allergic reactions can not be extrapolated and have not been provided by authors after requests. Previously reported grading system.
**Dunlop**	2018	Dunlop JH, Keet CA, Mudd K, Wood RA. Long-Term Follow-Up After Baked Milk Introduction. J Allergy Clin Immunol Pract. 2018:1699-1704.	wrong study design [Long-term follow-up of milk introduction]

**Dunn Galvin**	2018	Dunn Galvin A, Hourihane JO. Psychosocial Mediators of Change and Patient Selection Factors in Oral Immunotherapy Trials. Clin Rev Allergy Immunol. 2018;55(2):217-236.	different outcome [review on HR-QoL in Oral Immunotherapy trials]
**Furlong**	2001	Furlong TJ, DeSimone J, Sicherer SH. Peanut and tree nut allergic reactions in restaurants and other food establishments. J Allergy Clin Immunol 2001;108:867–870	self - reported diagnosis of food allergy
**Gabrielli**	2019	Gabrielli S, Clarke A, Morris J, et al. Evaluation of Prehospital Management in a Canadian Emergency Department Anaphylaxis Cohort. J Allergy Clin Immunol Pract. 2019 ;7(7):2232-2238.e3.	data referred to allergic reactions due to different allergenic triggers. Data on food-induced allergic reactions can not be extrapolated and have not been provided by authors after requests. Previously reported grading system.
**Gupta**	2008	Gupta RS, Kim JS, Barnathan JA, Amsden LB, Tummala LS, Holl JL. Food allergy knowledge, attitudes and beliefs: focus groups of parents, physicians and the general public. *BMC Pediatr*. 2008;8:36. Published 2008 Sep 19. doi:10.1186/1471-2431-8-36.	self-reported (parental report), not physician-diagnosed food allergy
**Gupta**	2019	Gupta RS, Warren CM, Smith BM, Jiang J, Blumenstock JA, Davis MM, Schleimer RP, Nadeau KC. Prevalence and Severity of Food Allergies Among US Adults. JAMA Netw Open. 2019 Jan 4;2(1):e185630.	self - reported physician-diagnosis of food allergy and corresponding reaction symptom-report
**Huang**	2012	Huang F, Chawla K, Järvinen KM, Nowak-Węgrzyn A. Anaphylaxis in a New York City pediatric emergency department: triggers, treatments, and outcomes. J Allergy Clin Immunol. 2012;129(1):162-8.e1-3.	data referred to allergic reactions due to different allergenic triggers. Data on food-induced allergic reactions can not be extrapolated and have not been provided by authors after requests. Previously reported grading system.
**Kilger**	2015	Kilger M, Range U, Vogelberg C. Acute and preventive management of anaphylaxis in German primary school and kindergarten children. BMC Pediatr. 2015;15:159.	data referred to allergic reactions due to different allergenic triggers. Data on food-induced allergic reactions can not be extrapolated and have not been provided by authors after requests. Previously reported grading system.

**Korenblat**	1999	Korenblat P, Lundie MJ, Dankner RE, Day JH. A retrospective study of epinephrine administration for anaphylaxis: how many doses are needed? Allergy Asthma Proc. 1999;20(6):383-6.	different outcome [no data referred to food allergy but only to anaphylactic reactions to inhalant allergen and hymenoptera venom immunotherapy and live hymenoptera stings]
**Nieto-Nieto**	2017	Nieto-Nieto A, Alonso MA, Farias-Aquino E, et al. Clinical Profile of Patients With Severe Anaphylaxis Hospitalized in the Spanish Hospital System: 1997-2011. J Investig Allergol Clin Immunol. 2017;27(2):111-126.	different otcome [prediction of severe anaphylaxis induced by different triggers]. No severy symptom score reported.
**Oropeza**	2017	Ruiz Oropeza A, Lassen A, Halken S, Bindslev-Jensen C, Mortz CG. Anaphylaxis in an emergency care setting: a one year prospective study in children and adults. Scand J Trauma Resusc Emerg Med. 2017;25(1):111.	data referred to allergic reactions due to different allergenic triggers. Data on food-induced allergic reactions can not be extrapolated and have not been provided by authors after requests. Previously reported grading system.
**Parker**	1990	Parker SL, Leznoff A, Sussman GL, Tarlo SM, Krondl M. Characteristics of patients with food-related complaints. J Allergy Clin Immunol. 1990;86(4 Pt1):503-11.	data referred to allergic reactions due to different allergenic triggers. Data on food-induced allergic reactions can not be extrapolated and have not been provided by authors after requests. No severy symptom score reported.
**Piromrat**	2008	iromrat K, Chinratanapisit S, Trathong S. Anaphylaxis in an emergency department: a 2-year study in a tertiary-care hospital. Asian Pac J Allergy Immunol. 2008;26(2-3):121-8	data referred to allergic reactions due to different allergenic triggers. Data on food-induced allergic reactions can not be extrapolated and have not been provided by authors after requests. The severity grading system is not clearly specified.
**Primeau**	2000	Primeau MN, Kagan R, Joseph L, et al. The psychological burden of peanut allergy as perceived by adults with peanut allergy and the parents of peanut-allergic children. Clin Exp Allergy. 2000;30(8):1135-1143.	self-reported (parental report), not physician-diagnosed food allergy
**Pumphrey**	2004	Pumphrey R. Anaphylaxis: can we tell who is at risk of a fatal reaction? Curr Opin Allergy Clin Immunol 2004; 4:285–90.	different outcome [review on prediction of fatal- anaphylaxis; no food allergy severity score reported]

**Samady**	2020	Samady W, Warren C, Wang J, Das R, Gupta R. Egg Allergy in US Children. J Allergy Clin Immunol Pract. 2020 May 3.	multiple publication
**Santaella**	2006	Santaella ML, Cox PR, Ramos C, Disdier OM. Anaphylaxis: an analysis of cases evaluated at the Puerto Rico Medical Center over a ten-year period. P R Health Sci J. 2006;25(2):143-7.	data referred to allergic reactions due to different allergenic triggers. Data on food-induced allergic reactions can not be extrapolated and have not been provided by authors after requests. Previously reported grading system.
**Sicherer**	2001	Sicherer SH. The impact of childhood food allergy on quality of life. Ann Allergy Asthma Immunol. 2001;87(6):461-464.	self-reported (parental report), not physician-diagnosed food allergy
**Tanno**	2017	Tanno LK, Chalmers RJ, Calderon MA, Aymé S, Demoly P; on behalf the Joint Allergy Academies. Reaching multidisciplinary consensus on classification of anaphylaxis for the eleventh revision of the World Health Organization's (WHO) International Classification of Diseases (ICD-11). Orphanet J Rare Dis. 2017;12(1):53.	different outcome [validation of ICD-11 codes for Anaphylaxis]
**Tanno**	2017	Tanno LK, Molinari N, Bruel S, Bourrain JL, Calderon MA, Aubas P, Demoly P; Joint Allergy Academies. Field-testing the new anaphylaxis' classification for the WHO International Classification of Diseases-11 revision. Allergy. 2017;72(5):820-826.	data referred to allergic reactions due to different allergenic triggers. Data on food-induced allergic reactions can not be extrapolated and have not been provided by authors after requests. Unclear reporting of grading system.
**Topal**	2019	Topal E, Bakirtas A, Yilmaz O, et al. Epidemiological and Clinical Features of Anaphylaxis: Single Center Experience with 109 Children. Pediat Aller Imm Pul.Jun 2013.88-92.	data referred to allergic reactions due to different allergenic triggers. Data on food-induced allergic reactions can not be extrapolated and have not been provided by authors after requests. Previously reported grading system.
**Turner**	2019	Turner PJ, Worm M, Ansotegui IJ, et al; WAO Anaphylaxis Committee. Time to revisit the definition and clinical criteria for anaphylaxis? World Allergy Organ J. 2019;12(10):100066. doi: 10.1016/j.waojou.2019.100066	Severity score is missing. Proposal for a new definition of anaphylaxis

**Wang**	2020	Wang HT, Warren CM, Gupta RS, Davis CM. Prevalence and Characteristics of Shellfish Allergy in the Pediatric Population of the United States. J Allergy Clin Immunol Pract. 2020 Apr;8(4):1359-1370.e2.	multiple publications
**Warren**	2020	Warren CM, Aktas ON, Gupta RS, Davis CM. Prevalence and characteristics of adult shellfish allergy in the United States. J Allergy Clin Immunol. 2019; 144(5):1435-1438.e5. doi: 10.1016/j.jaci.2019.07.031.	multiple publications

Table S3. Detailed characteristics of primary studies assessing symptom-specific severity of food allergy (n=23)

Study (First author, year of publication, country)	Study Design	Setting	Inclusion / exclusion criteria	Diagnosis of food allergy			Population	Outcome	Severity score used*	Results	Lesson learned
				positive sIgE	positive SPT	positive OFC					
Amin, 2012, USA	restrospec tive cohort	Tertiary center for allergic children	Pediatric cases from administrative routinely collected datasets in 2003 or 2008 were retrospectively included if the medical notes recorded a Dx of cow's milk and/or egg and/or fish and/or peanut and/or sesame and/or shellfish and/or soy and/or tree nut, and/or wheat allergy. Pts satisfying the criteria in both 2003 and 2008 were only recorded in the 2003 database.	+	+	NR	2003/2008:<br>• No. of included FA cases: 148 / 379<br>• Distinct pts, no. (%): 57 (3%) / 191 (8%)<br>• Male, no. (%): 106 (72%) / 242 (64%)<br>• Age at Dx (mean ± SD): 3.05 (±2.75) / 2.79 (±2.87) yrs<br>• Average sIgE at Dx, kU/L: 51.95 / 40.32 (p=0.002)<br>• Average SPT wheal, mm: 21 / 10<br>• Average SPT flare, mm: 20 / 22	• To compare prevalence & characteristics of FA in children referred to a tertiary care center in 2003 vs 2008	Amin, 2012	2003/2008<br>• FREQUENCY (from electronic medical chart) of ARs<br>Total no. of classified cases: 98 / 311<br>✓ Mild ARs, no. (%): 66 (67%) / 187 (60%)<br>✓ Moderate ARs, no. (%): 22 (23%) / 58 (19%)<br>✓ Severe ARs no. (%): 10 (10%) / 66 (21%)<br>• RECURRENCY OF AR: NR<br>EPINEPHRINE USE: NR<br>• No. OF PTs ADMITTED TO ED: NR<br>• No. OF PTs ADMITTED TO ICU: NR<br>• OTHER STATISTICAL ANALYSES:<br>Symptoms<br>Total no. of classified cases: 98 / 311<br>✓ Mucosal, no. (%): 10 (10%) / 62(20%)<br>✓ GI, no. (%): 8 (8%) / 64 (21%)	Authors observed an increase in the severity of ARs over a 5-year period accompanied by a decrease in the level of sIgE, which may be related to changing ethnical demographics

										✓ Respiratory, no. (%): 25 (26%) / 58 (19%)<br>✓ Vascular, no. (%): 2 (2%) / 16 (5%)<br>✓ Hives or angioedema, no. (%): 27 (28%) / 153 (49%)	
Astier, 2006, France & USA	Case-control	NR	NR	+	+	+/- DBP CFC	• 30 pts allergic to peanuts<br>✓Male, no.: 18<br>✓Age, mean±SD: 9.2±0.8 yrs (range, 3-20 yrs)<br><br>• 15 nonatopic subjects<br><br>• 15 pts allergic to birch pollen without FA.	To assess the performance for diagnostics and severity prediction of the 3 major recombinant peanut allergens [rAra h 1, rAra h 2, and rAra h 3]	Astier, 2006	• At DBPCFC in 17 peanut allergic pts:<br>✓ Pts with grade 1 AR, no. (%): 6 (35%)<br>✓ Pts with grade 2 AR, no. (%): 3 (18%)<br>✓ Pts with grade 3 AR, no. (%): 5 (18%)<br>✓ Pts with grade 4 AR, no. (%): 3 (29%)<br>• Self-reported accidental AR in 19 peanut allergic pts:<br>✓ Pts with grade 1 AR, no. (%): 3 (16%)<br>✓ Pts with grade 2 AR, no. (%): 6 (32%)<br>✓ Pts with grade 3 AR, no. (%): 3 (16%)<br>Pts with grade 4 AR, no. (%): 7 (37%)<br>• RECURRENCY OF AR: NR<br>EPINEPHRINE USE: NR<br>• No. OF PTs ADMITTED TO ED: NR<br>• No. OF PTs ADMITTED TO ICU: NR<br>• OTHER STATISTICAL ANALYSES:	Authors observed that sensitization established either by SPT or sIgE to rAra h 1 and/or rAra h 3 in addition to rAra h 2 is associated with more severe AR than monosensitization to rAra h2.

										✓ Neither SPT size nor levels of sIgE were correlated with AR severity.<br>✓ However, pts with monosensitization to rAra h 2 had a significantly lower severity score than polysensitized subjects (p<.02) and a lower level of sIgE against peanut extract and rAra h 2.	
Bernard, 2003, France, USA	Retrospective cohort	Tertiary center for allergic children	Several groups of children were selected. We focus on GROUP A = Hx strongly suggestive of peanut allergy (with $ occurring in the 30 min after one single ingestion of peanut) and +ve SPTs (unclear if consecutively recruited).	+	+	+/-	• 58 peanut allergic children met inclusion criteria for Group A.<br><br>• Detailed demographic characteristic are missing for GROUP A only.	To characterize the IgE response to a whole peanut protein extract, Ara h 1 and Ara h 2 in different groups of patients classified according to the severity of their AR	Sicherer, 1999	• FREQUENCY (at OFC)<br>✓ Pts with Mild ARs: n=16 (27.6%)<br>✓ Pts with Moderate ARs: n=16 (27.6%)<br>✓ Pts with Severe ARs: n=26 (44.8%)<br>• RECURRENCY OF AR: NR<br>• EPINEPHRINE USE: NR<br>• No. OF PTs ADMITTED TO ED: NR<br>• No. OF PTs ADMITTED TO ICU: NR<br>• OTHER STATISTICAL ANALYSES:<br>sIgE to whole peanut proteins (kU/l); Ara h 1 (kU/l); and Ara h 2 (kU/l):<br>✓ Pts with Mild ARs (n=16):<br>Mean 25.0; 2.1; 12<br>Max 158; 25; 100<br>Min 0; 0; 0	Authors suggested that level of peanut-sIgE could be used to avoid an OFC in the case of severe reactions. When compared to Ara h 1 and Ara h 2, whole peanut protein extract appeared to be the most appropriate allergen to perform the test.

										Median 7.5; 0.1; 1.35<br>✓ Pts with Moderate ARs (n=16):<br>Mean 84; 12.1; 43<br>Max 800; 120; 370<br>Min 0; 0; 0<br>Median 7.5; 0; 4.6<br>✓ Pts with Severe ARs (n=26)<br>Mean 179; 29; 122<br>Max 710; 140; 375<br>Min 1.8; 0; 0.3<br>Median 126; 12.5; 72<br>sIgE to Whole peanut proteins, Ara h 1 & Ara h 2 were each significantly higher in moderate or severe AR group vs mild AR (not in mild vs moderate AR)	
Boyano-Martìnez, 2009, Spain	Cross-sectional	Tertiary center for allergic children	All children 18 months or older who were given a Dx of IgE-mediated cow's milk FA in the clinical center and on a milk- and milk derivatives–free diet who presented for a regular clinic visit over a 16-month-long period were included. A questionnaire about possible ARs experienced in the last year was administered by the physician.	+	+	+/-	• 88 children allergic to milk (44 male; median age, 32.5 months) were recruited.<br>• COMORBIDITIES: 40 (46%) children had atopic dermatitis, 29 (33%) had asthma and 50 (57%) had other FA (egg, 47; seeds, 11; legumes, 8; and fish, 5).	• to calculate the frequency and severity of accidental exposure AR in children allergic to cow's milk during a 12-month period<br>• to identify risk factors for severe ARs.	Boyano-Martìnez, 2009	• RECURRENCY OF AR: 35 (40%) children reported 53 AR in the previous year: 21 children experienced only 1 AR; 12 pts 2 ARs, and 2 experienced 3 and 5 ARs, respectively.<br>✓ No. Mild ARs: n=28 (53%)<br>✓ No. Moderate ARs: n=17 (32%)<br>✓ No. Severe ARs: n=8 (15%)<br>• FREQUENCY<br>✓ Pts with Mild ARs: n=17 (49%)	ARs to accidental exposure are frequent in children with milk allergy. The proportion of severe ARs was 15%. The risk factors for such ARs included very high levels of sIgE to milk and casein and asthma

										✓ Pts with Moderate ARs: n=12 (34%)<br>✓ Pts with Severe ARs: n=6 (17%)<br>• TREATMENT: 30 (57%) ARs required pharmacologic treatment: antiH1 in 27 (51%), systemic steroids in 6 (11%), epinephrine in 4 (8%), and bronchodilator agents in 6 (11%).<br>• No. OF PTs ADMITTED TO ED: 11 pts<br>• No. OF PTs ADMITTED TO ICU: 1<br>• OTHER STATISTICAL ANALYSES:<br>✓ Median sIgE levels to cow's milk: significantly higher in children with severe ARs than in those with moderate ARs (37.70 vs 7.71 KUA/L, P.044) and mild ARs (3.37 KUA/L, P .009) or no ARs in the last year (3.89 KUA/L, P 5 .004).<br>✓ Analysis of data on sIgE levels to casein showed a similar association.<br>✓ Asthma: The frequency of severe ARs compared with moderate, mild, or no ARs was 10-fold higher in asthmatic children	

										(OR, 10.19; 95% CI, 1.13-91.54; P .022).<br>✓ A strong association was found between asthma and high sIgE levels to milk and casein. Asthmatic children have higher titers of sIgE to cow's milk than nonasthmatic subjects (median, 21.85 vs 2.87 KUA/L; P < .001). A similar association was found for sIgE levels to casein (median, 13.45 vs 2.26 KUA/L; P 5 .001). [Due to the low sample size unclear if asthma is an independent factor for having severe ARs or is just a modifier of the effect of sIgE levels]	
Boyano-Martìnez, 2012, Spain	Cross-sectional	Tertiary center for allergic children	All children 18 months or older who were given a Dx of IgE-mediated allergy to hen's egg, in the clinical center on egg and egg derivatives–free diet who presented for a regular clinic visit over a 16-month-long period were included. A questionnaire about possible ARs	+	+	+/-	• 92 children allergic to egg (55 male; median age, 52.2 months, range, 27-176 months) were recruited.<br>• COMORBIDITIES: 55 (60%) children had atopic dermatitis, 42 (46%) had asthma and 24 (52%) had other FA (fish, 21; seeds, 17; fruits, 15; legumes, 10; cow's milk, 6; and shellfish, 6).	• to calculate the frequency and severity of accidental exposure AR in children allergic to hen's egg during a 12-month period<br>• to identify risk factors for severe reactions.	Boyano-Martìnez, 2009	• RECURRENCY OF AR: 19 (21%) children reported 24 ARs in the previous year: 16 children experienced only 1 AR; 2 pts 2 ARs, and 1 experienced 4 ARs.<br>✓ No. Mild ARs: n=10 (42%)<br>✓ No. Moderate ARs: n=12 (50%)<br>✓ No. Severe ARs: n=2 (8%)<br>• FREQUENCY	ARs to accidental exposure are quite frequent in children with egg FA. The proportion of severe and moderate ARs was 48%.

			experienced in the last year was administered by the physician.							✓ Pts with Mild ARs: n=7 (37%)<br>✓ Pts with Moderate ARs: n=10 (53%)<br>✓ Pts with Severe ARs: n=2 (10%)<br>• <u>TREATMENT:</u> 9 (37%) ARs required pharmacologic treatment: antiH1 in 8 (33%), systemic steroids in 4 (17%), epinephrine in 0 pt. In 1 case this information was not known.<br>• <u>No. OF PTs ADMITTED TO ED</u>: 1 pt<br>• <u>No. OF PTs ADMITTED TO ICU:</u> 0<br>• <u>OTHER STATISTICAL ANALYSES:</u><br>✓ the risk of moderate or severe ARs was higher in children with lower total IgE titers (*P*<.05), children with higher titers of egg white sIgE, and younger children (differences near statistical significance).<br>✓ These results were similar when the risk of suffering moderate or severe ARs was compared with that of suffering mild or no ARs.	

										✓ the frequency of ARs was higher in children with higher titers of egg white sIgE (adjusted OR, 1.15; 95% CI, 1.03-1.28; P=.008) and in children with lower total serum IgE titers (adjusted OR, 0.16; 95% CI, 0.05-0.54; P=.001).	
Braganza, 2006, Australia	Case series	ED of a tertiary referral children hospital	All pediatric cases from administrative routinely collected dataset (Jul 1998-Jun 2001) were retrospectively included if the medical notes recorded a Dx of acute generalized allergic reactions triggered by any allergenic source<br><br>Previous Dx of food allergy in specialized centers: not reported	NR	NR	NR	583 cases met inclusion criteria (age range 0-14 yrs, median 4 yrs): mild/moderate (n=526) and severe allergic (n=57) reactions triggered by any allergenic source<br><br>Detailed demographic characteristic are missing for allergic reactions due to food allergens only.	• To assess incidence of paediatric anaphylaxis at an ED	Braganza, 2006	• FREQUENCY of ARs with a known food trigger (from electronic medical chart)<br>✓ Mild + moderate ARs: n=54 (62.8%)<br>✓ Severe ARs: n=32 (38.2%)<br>• RECURRENCY OF AR: NR<br>• EPINEPHRINE USE: NR‡<br>• No OF PTs ADMITTED TO ICU: 0<br>• OTHER STATISTICAL ANALYSES: Food items [No., Mild+Moderate / Severe ARs]<br>✓ Egg 11 / 7<br>✓ Dairy 6 / 8<br>✓ Peanut 10 / 3<br>✓ Other nut 7 / 3<br>✓ Fruit 9 / 0<br>✓ Seafood 5 / 3<br>✓ Composite foods 6 /8	Authors reported FA as the most frequent trigger for moderate and severe ARs in chidren.

Brown, 2004, Australia	Case-series	ED of a tertiary referral hospital for hymenoptera venom allergy	Cases from administrative routinely collected dataset (Oct 1990-Dec 1999) were retrospectively included if the medical notes recorded a Dx of a hypersensitivity or acute generalized allergic reactions triggered by any allergenic source<br><br>Previous Dx of food allergy in specialized centers: not reported	NR	NR	NR	• 1149 cases met inclusion criteria (age range 0-96 yrs, median 29 yrs).<br><br>• Food allergens are reported as a trigger in 18% (n= 205): sea food /n=47), nut (n=46), egg (n=14), monosodium glutamate (n=7), kiwi fruit (n=4), others or uncertain (n=87).<br><br>Detailed demographic characteristic are missing for allergic reactions due to food allergens only.	• To develop a simple grading system and definition of anaphylaxis.<br><br>• to identify predictors of reaction severity.	Brown, 2004	• FREQUENCY<br>✓ Pts with Mild ARs: n=96 (46.8%)<br>✓ Pts with Moderate ARs: n=93 (45.4%)<br>✓ Pts with Severe ARs: n=15 (7.3%)<br>• RECURRENCY OF AR: NR<br>• EPINEPHRINE USE: NR ǂ<br>• No OF PTs ADMITTED TO ICU: NR<br>• OTHER STATISTICAL ANALYSES: NR	Authors suggested that the moderate and severe grades may provide a workable definition of anaphylaxis.
Brown, 2013, Australia	Case-series	8 Australian EDs	Pts with a hypersensitivity or acute generalized allergic reactions triggered by any allergenic source were prospectively recruited between Jun 2006 and Feb 2009. Serial blood samples were collected.<br><br>Previous Dx of food allergy in specialized centers: not reported.	NR	NR	NR	• 412 cases in 402 pts met inclusion criteria (age range 3-99 yrs, median, 36 yrs; IQR, 24-50 yrs): mild (n=97) moderate (n=218) and severe allergic (n=97) reactions triggered by any allergenic source<br><br>• Food allergens are reported as a trigger in 131 cases (32%).<br><br>• Detailed demographic characteristic are missing for allergic reactions due to food allergens only.	• to define the clinical patterns of anaphylaxis<br><br>• to identify predictors of reaction severity.	Brown, 2004	• FREQUENCY, TOTAL No. ARs =131 (food only) + 6 (food + exercise)<br>✓ Mild ARs: n=19 (14.5%) / 1<br>✓ Moderate ARs: n=93 (71%) / 3<br>✓ Severe ARs: n=19 (14.5%) /2<br>1 pt died [allergen source (food?) NR]<br>• No. of Pt with ARs: NR(data reported cumulatively for different triggers)<br>• RECURRENCY OF AR: NR<br>• EPINEPHRINE USE: NR ǂ	Authors suggested that multiple inflammatory pathways drive reaction severity and support recommendations for safe observation periods after initial treatment.

										• <u>No. OF PTs ADMITTED TO ED</u>: NA<br>• <u>No. OF PTs ADMITTED TO ICU:</u> NR ǂ<br>• <u>OTHER STATISTICAL ANALYSES</u>:<br>• Analyses of severe reactions due to any allergic source were associated with older age, pre-existing lung disease, and drug causation.<br>• Subanalyses of allergic reactions due to food allergens only are missing.	
Clark, 2004, USA	Retrospective cohort	21 EDs in 9 US states and 4 Canadian provinces	All cases from administrative routinely collected dataset (Jan 1999 - Jan 2000) were retrospectively included if the medical notes recorded a Dx of allergic reactions triggered by food<br><br>Previous Dx of food allergy in specialized centers: not reported	NR	NR	NR	• A random sample of 678 charts (= 678 pts) was included from the 5296 charts identified<br>• Age (y), mean ± SD: 29 ± 18<br>• Female [% (95% CI)]: 57 (53-61)<br>• White [% (95% CI): 43 (38-47)<br>• Medical Hx of known allergy to food that caused current AR [% (95% CI): 41 (37-46)<br>• ALLERGIC COMORBIDITIES [% (95% CI): TOTAL No: 27 (24-30); Asthma:19 (16-22); Hay fever: 3 (2-4); Atopic dermatitis: 1 (1-3); Hives 1 (1-2); Angioedema 1 (0-2); Other allergic problems: 6 (4-8)<br>• Documentation of specific food in 92% (90-94) of pts) [% (95% CI)]:	• to describe the management of food-related acute allergic reactions	Clark, 2004	• <u>FREQUENCY</u>, TOTAL No. ARs = No. pts: n=678<br>✓Severe ARs (No [% (95% CI)]: 346 [51% (47-55)]<br>✓Other ARs [Mild + Moderate ARs (=pts with)]: n=332 (49%)<br>• <u>REATMENT</u>: Pts with severe ARS were more likely than those with less severe ARs to receive treatment with systemic steroids in the ED (54% vs 42%; *P* = .002), but this did not differ according to management with respiratory medications (36% vs 29%; *P* = .07). Only 24% of pts with severe ARs were treated with epinephrine in the ED.	In order to improve the concordance of the current approach to ARs in ED to guidelines, authors suggested to support a new collaboration between professional organizations in allergy and emergency medicine by developing educational programs and materials for ED patients and staff.

							Crustaceans 19 (16-22); Peanuts 12 (9-14); Fruits and vegetables 12 (10-15); Fish 10 (8-12); Tree nuts 9 (7-11); Milk 6 (4-8); Eggs 2 (1-4); Additives 1 (0.5-2); Other foods 36 (33-40)			• No. ADMISSION TO ICU: NR<br>• OTHER STATISTICAL ANALYSES: NR	
Corriger, 2019, France	Case-series	7 EDs in the Lorraine (northeast of France) urban region	Cases from administrative routinely collected dataset (Jan-Dec 2015) were retrospectively included if the medical notes recorded a Dx of allergic reactions triggered by any allergenic source<br><br>Previous Dx of food allergy in specialized centers: not reported	NR	NR	NR	323 cases (=pts) met inclusion criteria 67.2% adults (aged 18 to 88.4 yrs) and 32.8% children (age 2 mos to 18 yrs).<br><br>• 137 ARs in 137 pts. Culprit Food [children/adults/total, No.(%)]: peanut and nuts 29 (27.3%) / 9 (4.1%) / 38 (11.8%); Hen egg 6 (5.7%) / 1 (0.5%) / 7 (2.1%); Cow milk 9 (8.5%) / 0 (0.0%) / 9 (2.8%); Fish and meat 4 (3.8%) / 5 (2.3%) / 9 (2.8%); Shellfish 2 (1.9%) / 17 (7.8%) / 19 (5.9%); Other or unidentified 32(30.2%) / 23 (10.6%) / 55 (17.0%).<br><br>Detailed demographic characteristic are missing for allergic reactions due to food allergens only.	• To conduct the 1st multicenter epidemiological study in French emergency EDs in order to support ongoing national and international efforts for better Dx, management, and prevention of anaphylaxis	Ring, 1999	• FREQUENCY (from electronic medical chart) of ARs [total no. of pts with ARS=137],<br>✓ Pts with Mild ARs: n=24/137 (17.52%)<br>✓ Pts with Moderate ARs: n=47/137 (34.31%)<br>✓ Pts with Severe ARs: n=66/137 (48.17%)<br>• RECURRENCY OF AR: Biphasic ARs n=6/137 (4.38%); Previous history of anaphylaxis n=44/137 (32.12%)<br>• EPINEPHRINE USE: n=17/137 (12.41%)<br>• No OF PTs ADMITTED TO HOSPITAL (not ED): 26/137 (18.98%)<br>• No OF PTs ADMITTED TO ICU: 1/137 (0.73%)<br>• OTHER STATISTICAL ANALYSES: NR‡	Authors highlighted a urgent need for improved public health initiatives with respect to recognition and treatment of anaphylaxis.
Ewan, 2001, UK	Cohort	Regional specialist allergy clinic	All consecutive pts with Dx of peanut and/or nut allergy underwent detailed Hx on previous ARs to assess severity score and during	+	-	-	✓ 567 pts were followed prospectively.<br>✓Gender (M:F) = 1:1<br>✓Age at median onset of nut allergy 3 yrs (range 4 mos- 55 yrs), median	To assess a management programme providing advice on nut avoidance and emergency medication.	Ewan, 2001	• ARs before management (n=539) / Follow-up ARs (n=567), no. (%):<br>✓ grade 1 ARs: 130 (24%) / 50 (8.8%)	Authors highlighted that their management plan was effective, and their results indicate that

			follow-up with annual assessment. Pts, parents, school staff received verbal and written advice on nut avoidance and action plan for management of any AR				age at presentation 7.5 yrs (range 7 mos–65 yrs). 181 (32%) were under 5 yrs old, 193 (34%) were 5–11 yrs, 57 (10%) were 12–17 yrs, and 136 (24%) were older than 17 yrs.<br><br>✓351 (62%) pts were allergic to one nut only and 171 (30%) to more than one nut. 351 (62%) were allergic to peanuts, 85 (15%) to brazil nuts, 35 (6%) to hazelnut, 22 (4%) to both almond and walnut, 4 to pistachio, 2 to macadamia, and 1 to pine nuts. 130 (37%) of those allergic to peanuts were also allergic to a tree nut.<br><br>✓154 (21%) of 721 pts were lost to follow-up. These pts were similar to patients in the study group with respect to age at onset, age when seen at our hospital, severity of index reaction, nut type, sex ratio, and age distribution.			✓ grade 2 ARs: 56 (10.4%)/ 4 (0.7%)<br>✓ grade 3 ARs: 91 (16.9%)/ 8 (1.4%)<br>✓ grade 4 ARs: 191 (35.4%) / 23 (4.1%)<br>✓ grade 5 ARs: 71 (13.2%)/ 3 (0.5%)<br>88 (15%) of 567 pts had a ARs during follow-up (13 of 610 pt-months, median 21). Most pts with a moderate-severe index AR had a less severe follow-up reaction (p=0·017)<br>• <u>TREATMENT:</u> 26 pts had moderate to severe ARs (grade 4–5) during follow-up. Pts in this group were older (median 18 years *vs* 9 years; p<0·05). For moderate ARs, 5 of 23 needed oral antihistamines at most. 12 of 23 used an epinephrine inhaler, which was always successful. For moderate to severe ARs, 9 of 26 received an epinephrine injection.<br>• <u>No. OF PTs ADMITTED TO ED</u>: NR<br>• <u>No. OF PTs ADMITTED TO ICU:</u> NR	patients should be referred to specialist allergy centres for advice on nut avoidance.

										• <u>OTHER STATISTICAL ANALYSES:</u><br>✓ ARS BEFORE MANAGEMENT: The number of pts who had severe index ARs was 71 (13.2%) of 567. The median age of onset was 2 years for mild ARs and 11 years for moderate to severe ARs (p<0·005).<br>✓ FOLLOW-UP ARS: Most ARs were a result of snacks. The more severe ARs tended to occur during mealtimes (85% of ARs in restaurants were moderate to severe). The severity of the follow-up AR was related to the amount of nut ingested (p=0·012). Most pts reacted to a nut known to have caused a previous AR. 5% of pts developed an allergy to a nut that was previously tolerated. The prevalence of asthma in the series was 63% and 70% in pts who had a follow-up AR.	

Hourihane, 1997, UK	Cross-sectional	A validated[ϕ] diagnosis - questionnaire self-reported by peanut allergic subjects recruited all over UK evaluating the first and the last AR to peanut.<br><br>Mixed recruiting: Subjects comprised (a) a panel of 80 local people (mainly children) known to have peanut allergy and known to allergists together with other people referred by general practitioners for evaluation of suspected or proved peanut allergy; (b) people referred	Replies from a questionnaire self-reported by 833 peanut allergic subjects were reviewed by a single, experienced clinician. Only questionnaires (n=622), reporting typical symptoms and features of peanut allergy, were considered to have been submitted by people who were genuinely allergic to peanuts and included in the study.	+/- ϕ	+/- ϕ	+/- ϕ	622 (out of 833 having filled questionnaires) subjects (adult and children) who were judged to be genuinely allergic to peanuts by the questionnaire	• To determine the patterns of clinical severity, $ progression and management of ARs in a large group of peanut allergic subjects.	Hourihane,1997	• FREQUENCY (self-reported[ϕ]) Comparison of first and most recent reactions in 527 subjects (data missing on 10 subjects, 85 subjects one reaction only): Mild /mild no.=44; mild/moderate: 29; mild/severe: no.=34; moderate /mild no.=27; moderate/moderate no.=111; moderate/severe no.=59; severe/mild no.=21; severe/moderate no.=29; severe/severe no.=173<br>The most severe $ (collapse and cyanosis) were reported by only 42 subjects (7%) on first exposure and 46 (7%) subjects on most recent AR.<br>• RECURRENCY OF AR: Excluding 85 subjects who had only 1 AR to peanuts, 33 (6%) had an AR to peanuts in the previous month; 51 (9%) had not had a AR to peanut for more than 5 years	Authors observed that SPT and peanut sIgE levels do not predict clinical severity

		through the Anaphylaxis Campaign (a national charity); and (c) people who contacted the study coordinator after having seen details of the study in national newspapers and magazines.								• EPINEPHRINE USE: 78 pts (15%) received or self-administered injected adrenaline to treat the most recent AR. These subjects had varying combinations of minor and major $. Adrenaline was administered to 60 of 247 subjects with wheeze (24%), 28 of 54 subjects with cyanosis (52%) and 18 of 43 (41%) of subjects with collapse. Of the 78 adrenaline injections administered 68 (82%) were given within 1 h and 72 (92%) within 2h.<br>• No OF PTs ADMITTED TO ED: 232 pts (37%) had never attended hospital because of AR to peanuts and only 82 (35% of hospital attenders, 13% of entire group) had been admitted overnight.<br>• No OF PTs ADMITTED TO ICU: 2 (0.3%).<br>• Severe ARs on most recent AR correlated strongly with both hospital admission (x^2	

										=2.03, P<0.01, OR= 3.7) and the administration of injected adrenaline (x^2 = 21.36. P<0.0001, OR 2.87).<br>• OTHER STATISTICAL ANALYSES:<br>✓ AGE: Mild ARs were more common in children and severe ARs more common in adults (Kruskal-Wallis one-way ANOVA, P = 0.0002)	
Hourihane, 2005, UK	Cross-sectional	Cross-sectional questionnaire assessment of community-based ARs and low-dose DBPCFC in self-selected peanut-allergic volunteers from local adult and paediatric allergy clinics and national advertisements.	The inclusion criteria were: 6-66 yrs of age; a convincing Hx of an AR to peanut in the last 3 yrs before OFC and +ve SPT; consent to DBPCFC	+	+	+ DBP CFC	• 40 challenged volunteers allergic to peanut (out of those 151 who agreed to complete questionnaires only)<br><br>✓GENDER [Child; Adult; Total, M:F] 8:15; 7:10; 15:25<br>✓Children, no.=23<br>✓Asthma [Child; Adult; Total, No.]: 13; 10; 23<br>✓Eczema [Child; Adult; Total, No.]: 12; 9; 21<br>✓Rhinitis [Child; Adult; Total, No.]: 11; 10; 21<br>• 23 patients were asthmatic<br>• reported allergies to other foods	To explore the relationship of a subject's Hx of past ARs to the severity of AR elicited by a low-dose, DBPCFC with peanut.	Hourihane,2005	• At DBPCFC in 40 peanut allergic pts:<br>✓ Pts with Mild ARs: n=9 (22.5%)<br>✓ Pts with Moderate ARs: n=22 (55%)<br>✓ Pts with Severe ARs: n=4 (10%)<br>✓ 5 pts had no ARS<br>• RECURRENCY OF AR: Self-reported previous accidental AR in 40 peanut allergic pts: n=3 (range 1–20).<br>• EPINEPHRINE USE: 8 pts (21%) had used their autoinjectors in previous ARs experienced in the community.<br>• No. OF PTs ADMITTED TO HOSPITAL: 11 pts (27%) had attended hospital after their	Authors proposed to combine dosage and symptom grades to give an overall score for each community reaction and the reaction elicited by low-dose DBPCFC

										most severe ARs to peanut<br>• OTHER STATISTICAL ANALYSES:<br>✓ There was no difference in the challenge score between age groups, gender and asthma status using the Mann–Whitney U-test<br>✓ There was no apparent correlation between the mean diameter of peanut SPT weal and PsIgE concentration. Within the paediatric population, there was a weak association between PsIgE and SPT size (Spearman's rank correlation<br>✓ R=0.40, P=0.04, n=23).	
Itazawa, 2020, Japan	Case-series	Cross-sectional questionnaire assessment of community-based ARs and low-dose DBPCFC in self-selected peanut-allergic volunteers from 142 local adult and paediatric	Pts (up to 18 yrs of age) with convincing Hx of FA and consent to OFC to the culprit food. EXCLUSION CRITERIA: pts older than 19 yrs of age, pts who had been administered placebo Food or had undergone food-exercise challenges, or had insufficient	+	+	+	• 5062 pts (median age, 3.8 years;<br>males, 65.2%) were included and performed OFC. Reasons for OFC: 777 (15.3%) were for confirming Dx; 2408 (47.6%) for confirming tolerance, 1501 (29.7%) for determining safe intake quantity, and 376 (7.4%) for assessing threshold level for OIT.	To explore the relationship of a subject's Hx of past ARs to the severity of AR elicited by a low-dose, DBPCFC with peanut.	Itazawa, 2020	• FREQUENCY: At OFC 2258 (44.6%) pts presented ARs:<br>✓Pts with grade 1 ARs: n=991 (43.9%)<br>✓Pts with grade 2 ARs: n=736 (32.6%)<br>✓Pts with grade 3 ARs: n=340 (15.1%)<br>✓Pts with grade 4-5 ARs: n=190 (8.5%)<br>✓Pts with grade 4-5 ARs: n=1 (0.04%)	Authors suggested that prevalence, severity, and treatment of AR differ depending on the indication for OFC.

		allergy clinics and national advertisements between Mar 2012 and May 2013.	questionnaire information.							• Positive OFCs were seen in 2258 (44.6%) pts with the following prevalence by food type: hen's egg, 43.5% (n = 1074); cow's milk, 54.6% (n = 605); and wheat, 55.0% (n = 330)<br>• <u>RECURRENCY OF AR</u>: NR<br>• <u>EPINEPHRINE USE</u>:<br>✓Pts with grade 1 ARs: n=0<br>✓Pts with grade 2 ARs: n=30<br>✓Pts with grade 3 ARs: n=45<br>✓Pts with grade 4-5 ARs: n=84<br>✓Pts with grade 4-5 ARs: n=1<br>• <u>No. OF PTs ADMITTED TO ICU</u>: NR<br>• <u>OTHER STATISTICAL ANALYSES:</u><br>✓ The severity of ARs differed significantly depending on the type of indication for OFC. Adjusted standardized residuals demonstrated that the prevalence of grade 1 severity was highest for the indications to confirm Dx and tolerance, and the prevalence of grade 3	

										and grade 4-5 severity was highest for assessing threshold level for OIT.<br>✓ In addition, the use of epinephrine was higher for the indication to determine safe intake quantity than for the other indications.	
Macdougall, 2002, UK	Cohort	Primary, secondary, tertiary care, population-based the offices of national statistics	The following databases searched: notifications of death from the offices of National statistics, The British Pediatric Surveillance Unit (BPSU); Database of allergy death held by Dr. R Pumphrey; Anaphylaxis Campaign, Personal letters to 10 pediatric allergy specialists; Asthma and Allergy Information Research; Daily Telegraph and The Times stored on CD-ROM; pediatricians	-	-	-	A retrospective search for fatalities in children 0-15 yrs between 1990-1998, primarily of death certification at offices of national statistics. A prospective survey of fatal and severe reactions from March 1998 to February 2000, primarily through BPSU.	The incidence of severe and fatal allergic reactions to food	Macdougall, 2002	• FREQUENCY:<br>✓ Deaths: 0.006 deaths per children 0-15 year-old per year<br>✓ Severe ARs: 49 pts (10 caused by peanut)<br>✓ Near fatal ARs: 6 pts (requiring intubation)	If 5% of children have food allergy, the risk that a food allergic child will die from a food induced allergic reaction is about 1 in 800 000 per year. Asthma may be a risk factor for these ARs
Moro-Moro, 2011, Spain	Retrospective cohort	different clinical settings of the catchment area of Hospital	Cases of anaphylaxis were retrieved from the databases of ED medical notes recorded a Dx of	NR	NR	NR	213 anaphylaxis cases (age range 0 to > 69 yrs) met inclusion criteria. Food was as a trigger in 28.6% cases (62 pts).	Incidence of anaphylaxis and subtypes of anaphylaxis in an ED	Brown 2004	• FREQUENCY: 62 pts had moderate-severe ARs<br>✓ Pts with Mild ARs: NA<br>✓ Pts with Moderate ARs: 53 (85.48%)	From 213 anaphylaxis cases food was as a trigger in 28.6% cases

		Universitario Fundación Alcorcón (HUFA), Alcorcón, Spain	anaphylaxis or any acute generalized allergic reactions triggered by any allergenic source Previous Dx of food allergy in specialized centers: not reported				Detailed demographic characteristic are missing for allergic reactions due to food allergens only.			✓ Pts with Severe ARs: 9 (14.52)<br>• RECURRENCY OF AR: NR<br>• EPINEPHRINE USE: NR<br>• No OF PTs ADMITTED TO ED: NR	
Primeau, 2000, Canada	Case-control	Mixed recruitment through reviewing charts of pts referred to the allergy clinics of the Montreal Children's hospital or the Montreal General Hospital between January 1993 and December 1997; consecutive peanut-allergic pts presenting to the allergy clinics; advertising through a lay educational/support organisation; newspaper	Peanut-allergic children and adults compared vs children and adults with rheumatological disease	NR	NR	NR	153 peanut-allergic (PA) children were compared with 69 children with a rheumatological disease (RD); 37 PA adults with 42 adults with RD<br><br>ALLERGIC COMORBIDITIES: Eczema: PA children =57%; PA adults = 35%; allergic rhinitis: PA children =36%; PA adults=68%; other food allergies: PA children =359; PA adults=86%	To compare the QoL and family relationships of children and adults with PA to that of children and adults with RD	Primeau, 2000	• FREQUENCY (patient/parent self-report on the severity of the first AR)<br>✓ Pts with Mild ARs: PA children 38%; PA adults=52%<br>✓ Pts with Moderate ARs: PA children= 51%; PA adults=48%<br>✓ Pts with Severe ARs: PA children = 11%; PA adults=0%<br>• RECURRENCY OF AR: NR<br>• EPINEPHRINE USE: NR<br>• No OF PTs ADMITTED TO ED: NR<br>• No OF PTs ADMITTED TO ICU: NR	The psychological burden of PA as perceived by adults with PA and the parents of PA children shows the need to support these families.

		advertisements.									
Tejedor-Alonso, 2013, Spain	Retrospective cohort	different clinical settings of the catchment area of Hospital Universitario Fundación Alcorcón (HUFA), Alcorcón, Spain	Cases of anaphylaxis were retrieved between 1998 and 2005 from the databases of ED, hospitalized pts, and primary care centers if the medical notes recorded a Dx of anaphylaxis or any acute generalized allergic reactions triggered by any allergenic source<br>Dx of food allergy was confirmed by experienced allergist physicians	NR	NR	NR	1512 pts of all ages (mean, IQR] age, 34.9 [12-49] yrs; 57.8% women) experienced anaphylaxis due to different triggers.<br><br>337 pts out of 1512 had food induced anaphylaxis.<br><br>Detailed demographic characteristic are missing for allergic reactions due to food allergens only.	To evaluate the incidence of first recurrence of anaphylaxis and examine the risk factors associated with recurrence.	Brown 2004 (definition of anaphylaxis : Sampson 2006)	• <u>FREQUENCY:</u> Total number: 880 ARs out of 2510 cases of anaphylaxis due to different triggers<br>✓ Mild ARs, no. (%): 0 (0%)<br>✓ Moderate ARs, no. (%): 801 (91.02%)<br>✓ Severe ARs, no. (%): 79 (8.98%)<br>• <u>RECURRENCY OF AR</u>: 18 pts had recurrent ARs (31.58%)<br>• <u>EPINEPHRINE USE:</u> 44 cases<br>• <u>No. OF PTs ADMITTED TO ICU:</u> NR	
Van Erp, 2013, The Netherlands	Retrospective cohort	Tertiary hospital	Cases were collected retrospectively from Electronic Patient Records of DBPCFC , conducted in the Wilhelmina Children's Hospital during 3-yr period	+	+	+ DBPCFC	225 DBPCFCs for peanut; median age (IQR), in yrs) – 6.7 (5.0-9.5); male 148 (66%)<br><br>In 109 (48%) children, positive DBPCFC<br><br>ALLERGIC COMORBIDITIES:<br>Allergic rhinitis= 96 (43%);<br>Asthma using ICS=83 (37%);<br>Asthma using inhalants=102 (45%);<br>Other food allergy=151 (67%).	To determine predictors for positive and severe OFC outcome	Sampson, 2003	• <u>INCIDENCE [DBPCFC outcome]</u><br><u>No. of pts (=No. ARS) with positive OFC</u>: 109 (48%)<br>✓ Pts with Mild/ Moderate ARs: n=85<br>✓ Pts with Severe ARs: n=24<br>• <u>RECURRENCY OF AR</u>: Pts who reported previous severe AR:<br>✓ among those with positive DBPCFC: n=24<br>✓ among those with severe ARs at DBPCFC: n=6	The level of sIgE, male gender and having another food allergy were independently related to positive OFC. The final model showed good discrimination of children with positive and negative OFC with AUC of 0.89 (0.84-0.93), but calibration was poor.<br>None of the studied risk factors could

									• EPINEPHRINE USE (IM or IV) during DBPCFC in 16 pts (15%) with:<br>✓ Mild/moderate AR: n=1 (1%);<br>✓ Severe AR (15 (63%)<br>• No OF PTs ADMITTED TO ED: NR<br>• No OF PTs ADMITTED TO ICU: NR<br>• OTHER STATISTICAL ANALYSES:<br>DISCIMINATION: ROC area (95% CI)= for adjusted OR for positive OFC (95% CI)=0.89 (0.84-0.93)<br>CALIBRATION: Hosmer-Leme show (chi-squared statistic) for adjusted OR for positive OFC (95% CI)=34.99 (df=8), p<0.005	predict a severe AR during DBPCFC.
Van Erp, 2014, The Netherlands	Retrospective cohort	An academic hospital	All pts who completed DBPCFC (n=191) for peanut performed between 2008-2010 in an academic hospital in the Netherlands	+	NR	+	Children (n=191) aged between 3.4-18.6, mean 7.8 yrs old; male 132 (70%); peanut sIgE, median (IQR) in kU/l – 2.60 (0.60-18.80)	To assess inter and intra-observer variability in interpretation of clinical symptoms during DBPCFC	Sampson, 2003	• FREQUENCY [DBPCFC outcome]<br>No. of pts (=No. ARS) with positive OFC [No. (%)]: 88 (46%)<br>✓ Grade 1: 2 (1%)<br>✓ Grade 2: 51 (27%)<br>✓ Grade 3: 15 (8%)<br>✓ Grade 4: 20 (11%)<br>• RECURRENCY OF AR: NR<br>• EPINEPHRINE USE: NR<br>• No OF PTs ADMITTED TO ED: NR	There are considerable amount of variability in reassessment of $ recorded during DBPCFC sheets between and within well trained clinicians, particularly when subjective $ occur

										• No OF PTs ADMITTED TO ICU: NR<br>• OTHER STATISTICAL ANALYSES: NR	
Vetander, 2014, Sweden	Retrospective cohort	Secondary care; Paediatric hospitals	Cases from medical records of all 358 children with ED visits due to allergic reactions to food at three paediatric hospitals in Stockholm County, Sweden between 1 January 2007 and 30 June 2010.<br><br>Previous Dx of food allergy in specialized centers: not reported	-	-	-	Children with no ED revisits (n=278; mean age=5.7 (SD=5.5); girls 129 (46%); Children with ED revisits (n=80; mean age=6.1 (SD=4.9); girls 40 (50%).<br><br>ALLERGIC COMORBIDITIES: Asthma 37 (46%); eczema 33 (41%); allergy to inhalant allergens 36 (45%); previously known food allergy – 1 food allergy – 25 (31%); 2 and more food allergies 49 (61%)	To evaluate the incidence and potential risk factors for repeated ED visits for food-allergic reactions among children with a prior ED visit due to reactions to food	Vetander, 2011 [Vetander M, et al. Classification of anaphylaxis and utility of the EAACI Taskforce position paper on Anaphylaxis in Children. Pediatric Allergy Immunology 2011; 22: 369–373]	• FREQUENCY: incidence rate of ED revisits of 9 per 100 patient-years. Children with no ED revisits (n = 278)/Children with ED revisits (n = 80), index AR/ Children with ED revisits (n = 80), ARs at ED revisit [No. of children (%)]<br>✓ Mild- Moderate ARs: 79 (28%) / 27 (34%) / 16 (20%)<br>✓ Severe ARs: 12 (4%) 6 (8%) 4 (5%)<br>• RECURRENCY OF AR: ARs at revisit (%): 21% severe ARs; 38% less severe; 41% comparable severity.<br>• N° ARS TO THE CULPRIT FOOD: Any tree nut-peanuts: 28 (38%); egg 10 (13%); milk 10 (13%); other foods 15 (19%); unknown 17 (21%)<br>• EPINEPHRINE USE: 23 (40%) had adrenaline at the time point for the ED revisit	The severity of the ARs at the ED revisit could not be predicted by the severity of the index AR (unpredictability of food-induced allergic reactions).Previously known FA and prior prescription of adrenaline are significant risk factors for ED revisits among children with a prior ED visit due to AR.

										• <u>No OF PTs ADMITTED TO ICU:</u> NR<br>• <u>OTHER STATISTICAL ANALYSES:</u><br>✓ Known FA before the index-visit in 2007 was identified as a risk factor for ED revisits (RR = 2.30, 95% CI 1.35–3.94) with a tendency towards higher risk among children with two or more food allergies.<br>✓ Prescription of adrenaline autoinjector before the index-AR also emerged as a risk factor for ED revisits (RR = 2.02, 95% CI 1.17–3.49). Additional adjustment for asthma and allergy to inhalant allergens did not change the results.<br>✓ Allergy to inhalant allergens before the index-reaction showed a tendency towards increased risk of ED revisits (RR = 1.73, 95% CI 0.97–3.10).<br>✓ Having a more severe index-reaction seemed to be	

										positively associated with the risk for ED revisits, however, without reaching statistical significance most likely due to limited number of observations.	
Virkud, 2019, USA	Retrospective cohort	Secondary-tertiary care	Cases from Massachusetts General Hospital allergy practices who underwent OFC for suspected almond allergy	+	+	+	590 pediatric and adult pts, aged 1 to 66 years who had been referred for OFC (open) for suspected almond allergy from 2009 to 2018.<br><br>Female 249 (42.3%);<br><br>Race: white 461 (83.2%); black or African-american 7 (1.3%); Asian 57 (10.3%); Hispanic-latino 26 (5.8%)	To evaluate almond oral challenge outcomes and assess the predictive value of clinical testing	AR grading system: Niggemann 2016<br><br>Anaphylaxis definition: Sampson 2006	<u>FREQUENCY (OFC)</u><br>✓ Total No. OFC=590<br>✓ -ve OFC=545 (92%);<br>✓ Indeterminate OFC outcome=15(3%)<br>✓ +ve OFC=30 (5%)<br>• <u>Among the 30 positive OFC:</u><br>✓ AR grade 2/3: n = 21 (4%)<br>✓ Anaphylaxis: 3 (0.5%)<br>• <u>RECURRENCY OF AR</u>: 11 subjects had multiple challenges to almond, comprising 24 OFCs total. Ten pts initially had +ve or indeterminate OFCs and then eventually went on to pass the final OFC. The median time to rechallenge was 2.7 years (mean, 2.3 years). Most subjects had mild $. Only 1 subject passed an OFC and then went on to fail an OFC.<br><u>EPINEPHRINE USE [No. (%):</u><br>✓ Among pts with +ve OFC (n=21): 2 (7%);	Almond-specific IgE level, SPT weal diameter and age at challenge combined resulted in good predictive value for grade 2/3 ARs by receiver-operating characteristic analysis (AUC – area under curve, 0.83). Anaphylaxis is possible with high almond sensitisation.

										✓ Among pts with grade 2/3 ARs (n=21): n= 2 (10%);<br>✓ Anaphylactic AR (n=3): 2 (67%)<br>• No OF PTs ADMITTED TO ED: NA<br>• No OF PTs ADMITTED TO ICU: 0<br>• OTHER STATISTICAL ANALYSES: Grade 2/3 ARs: SPT+IgE+Age: AUC 0.83	
Ye, 2015, Korea	Retrospective cohort	Secondary care	Cases from medical records on adult pts diagnosed with anaphylaxis due to different allergenic triggers in 15 University Hospitals of South Korea.<br><br>Dx of food allergy in specialized centers: not reported	-	-	-	A total of 1,806 cases (52% male, age 16-86 yrs) with Dx of anaphylaxis between 2007 and 2011 in 15 University Hospitals of South Korea. Anaphylaxis to food 430 pts (24.2%); seafood 145 (8.2%); wheat 147 (8.3%); nuts 29 (1.6%); meats 56 (3.2%); vegetables 58 (3.3%)	To investigate the causes and clinical features of anaphylaxis; predictor factors of the severity and serious outcomes of anaphylaxis in Korean adults	Brown, 2004	• FREQUENCY:<br>✓ Mild AR (n=72): seafood 28 (8.2%): wheat 16 (4.7%); nuts 3 (0.9%); meats 7 (2.1%); vegetables 13 (3.8%)<br>✓ Moderate AR (n=176): seafood 61 (8.9%): wheat 60 (8.7%); nuts 12 (1.7%); meats 24 (3.5%); vegetables 21 (3.0%)<br>✓ Severe AR (n=182): seafood 56 (7.5%): wheat 71 (9.5%); nuts 14 (1.9%); meats 25 (3.4%); vegetables 24 (3.2%)<br>• RECURRENCY OF AR: NR ǂ<br>• EPINEPHRINE USE: NR ǂ	Food was not a predictors of serious outcomes, including prolongation of admission, or new admission, for anaphylaxis OR (95% CI) 0.978 (0.571-1.678), P<0.937. However, wheat consumption was one of the predictors of severe anaphylaxis (2.425; 95% CI 1.054-5.581), P<0.037.

										• No OF PTs ADMITTED TO ED: NR ǂ<br>• No OF PTs ADMITTED TO ICU: NR ǂ<br>• OTHER STATISTICAL ANALYSES: NR ǂ	

List of abbreviations: AR, food induced - allergic reaction; CI, confidential interval; DBPCFC, double-blind placebo-controlled food challenge; Dx, diagnosis; ED, emergency department; EoE, eosinophilic esophagitis; EPR, Electronic Patient Records; FA, food allergy; FPIES, food protein-induced enterocolitis; s Hx, clinical history; ICU, intensive care unit; IQR, interquartile range; NR, not reported; OFC, oral food challenge; PA, peanut allergy; RD, rheumatological disease; sIgE, specific Immunoglobulin E; SPT, skin prick test; $, symptom(s); +ve, positive.; -ve, negative.

*Detailed characteristics of symptom severity scoring systems are described in **Table 1.**

ǂ data reported cumulatively for different triggers.

ɸ [*Hourihane, 1997*] A subgroup of 69 adults underwent SPT and OFC. This group revealed a 13% false positive rate (9 out of 69) for questionnaire-based diagnosis of peanut allergy in adults. Therefore, questionnaire had been validated with a sensitivity of 100% and a specificity of 87% for detecting peanut allergy compared with the gold standard of DBPCFC.

Table S4. Detailed characteristics of primary studies assessing Food Allergy related-Quality of life measures (n=7)

**Study** (First author, year of publication, country)	**Study Design / Methodology**	**QoL form**	**Diagnosis**	**Domains**	**Population Groups**	**Outcome**	**Results**	**Lesson learned**
Dantzer, 2018, US	Cross-sectional<br><br>All participants referred by specialist	Food Allergy Quality of Life Questionnaires Parent Proxy Form (FAQLQ-PF)<br>Child Form (FAQLQ-CF) and Teenager Form (FAQLQ-TF)	Based on the combination of history, skin testing, and IgE results.	Allergen avoidance ; Risk of accidental exposure (RAE);<br>Emotional impact (EI);<br>Food Anxiety (FA);<br>Social & Dietary Limitations (SDL)	N =58 families (group 1: 37, group 2: 21)	To determine the impact of tree nut OFCs and selective nut avoidance on FAQL and risk of reactions.<br>Tree nut OFCs were most often offered to patients estimated to have a high chance of a successful outcome.<br>Group 1 (pts who chose to undergo select tree nut OFCs) vs Group 2 (pts who chose complete nut avoidance).	FAQLQ-PF showed no clinically meaningful difference between groups 1 and 2.<br>FAQLQ-CF showed higher scores in group 1 versus group 2 (minimal clinical important difference (MCID), defined as the smallest difference that patients perceive as beneficial, MCID <0.45)<br>TF opposite results with lower overall, EI, and RAE in group 1 versus group 2 (MCID <0.45)<br>All respondents answered yes to the question, “Overall, has your QoL improved since the tree nut OFC?”	Tree nut OFCs may be beneficial in clarifying a diagnosis, decreasing uncertainty, and expanding the diet. If the specific tree nuts to be challenged are chosen carefully, the procedure is extremely safe (93% success rate).<br>Introduction of tree nuts did not increase the rate of accidental peanut or tree nut reactions<br><br>The benefits of selected TN challenges, especially with regard to FAQOL, appear to outweigh the risks.<br><br>Post-challenge data limited by short follow-up time and small sample size.
DunnGalvin, 2010, Ireland and The Netherlands	Longitudinal, psychometric measurement (3 time points pre/post- OFC)<br><br>All participants referred by specialist	Food Allergy Quality of Life-Parent Proxy Form (FAQLQ-PF) and the Food Allergy Independent Measure (FAIM)	Food Challenge outcome. Symptoms reported Reactions during OFC	Emotional Impact (EI);<br>Food Anxiety (FA);<br>Social & Dietary Limitations (SDL);<br>Expectation of outcome if allergen	Parents (n=84) of children 0–12 years undergoing OFC<br><br>Group A (positive OFC) and	To evaluate longitudinal validity, differences between Group A (positive challenge) and Group B (negative challenge) were expected over time.<br>To determine the minimally important difference (MID).	Sensitivity and responsiveness demonstrated by significant differences between positive and negative groups at 6 months [F = 6.221, P=0.003].<br>Anaphylaxis (OR 2.112) and severe symptoms (OR 2.221) and number of foods avoided (OR 1.396),	FAQLQ-PF is responsive to change in a food-allergic patient population with disease-specific clinical outcomes.<br>FAQL improved for both groups (more improvement for OFC negative group) showing that OFC may have a therapeutic effect even if OFC positive due to a

				accidentally ingested.	Group B (negative OFC)		predicted 'no improvement' at endpoint. MID was 0.45 on a seven-point response scale.	reduction in uncertainty (also see comment re 'Van der Velde 2012' paper below)<br><br>More adverse impact on FAQLQ according to severity, challenge result, number of symptoms, recent anaphylaxis (<6 months)
Flokstra-de Blok, 2010, Ireland and The Netherlands	Cross-sectional psychometric study<br><br>The patients were recruited at an outpatient allergy clinic, based on a convincing history of food allergy supplemented by analysis of specific IgE to the foods in question.	Generic questionnaires (CHQ-CF87 and RAND-36) and disease-specific (FAQLQ-CF, -TF and -AF)	Physician-diagnosed food allergy. Severe food allergy was defined as having a prescription for an epinephrine auto injector (EAI), or self-reported previous episodes of anaphylaxis (i.e. the symptoms "difficulty breathing", "inability to stand", collapse and/or loss of consciousness).<br><br>Self-report	Generic physical functioning, role functioning-emotional/behaviour/physical, general behaviour, mental health, self-esteem, family activities /cohesion<br><br>FA-specific Allergen avoidance; Risk of accidental exposure (RAE);Emotional impact (EI); Dietary restrictions	79 children, 74 adolescents and 72 adults with food allergy.	To compare HRQL of food allergic patients as measured with generic and disease-specific questionnaires<br>Floor and ceiling effects, percentage of agreement.<br>Anaphylaxis (yes/no), symptom groups, and association with FAQLQ also examined	FAQLQs showed minimal floor or ceiling effects. Generic- minimal floor effects, but very high ceiling effects (>73%).<br>Low percentages of agreement between generic and disease-specific questionnaires to identify the same food allergic patients with the best or worst HRQL<br>FAQLQ-AF scores among patients who reported anaphylactic reactions (n = 43) compared to those who did not (n = 36), did not reach statistical significance, although trend (5.04 vs. 4.63 p = 0.10). When using EQ-5D, no differences were found between patients with and without self-reported anaphylaxis, (mean EQ-5D index values 0.81 vs. 0.79, respectively).	Disease-specific HRQL questionnaires are more suitable to measure clinically important impairments in HRQL or HRQL differences over time in food allergic patients.<br>Generic HRQL questionnaires needed for comparison between different diseases.

							Regression coefficients were significant for the OAS and cardiovascular symptoms.	
Goossens, 2011, the Netherlands and US	Cross-sectional/translation<br><br>Online questionnaires	FAQLQ-AF (Food allergy quality of life questionnaires-Adult Form)<br><br>And FAIM (which measures expectation of outcome/perception of severity)	Self-report on list of symptoms		American adults with FA (N=180)	FAQLQ-AF translated from Dutch into English (cross cultural validity)	Good construct validity (correlation with FAIM: r = 0.72; p<0.001), internal consistency (Cronbach's a = 0.95) and discrimination<br>Significantly greater FAQL impairment in US vs Dutch (4.3 vs. 3.5, respectively) p<0.001<br>Discriminative ability was shown for experiencing anaphylaxis vs. not experiencing anaphylaxis in both American participants (5.4 vs. 4.9; P = 0.03) and Dutch participants (4.68 vs. 3.60; P = 0.01)<br><br>The total FAIM scores in the American participants were also significantly higher than in Dutch participants<br>(4.4 vs. 3.9; P = 0.00).	HRQL of American food-allergic adults more impaired than Dutch food-allergic adults.<br>Issues such as knowledge, attitudes and beliefs may differ between cultures and could possibly have accounted for the differences in HRQL.<br>Other cultural differences may include eating out practices and amount.<br><br>Limitations: Biases resulting from the differences in participant recruitment.<br>Four items of the FAQLQ-AF showed an insufficient correlation with the total FAIM score
Tackett, 2018, US	Cross-sectional online survey<br><br>Participants were 183 caregivers (50% mothers) who had a child with physician-diagnosed food allergies	Caregivers completed:<br>a set of online questionnaires about food insecurity (FIS), use of food assistance programs (SNAP, food banks), and history of FIA	Self-report<br>Screened for answer to question – does your child have physician diagnosed FA	Uncertainty or inability to meet family food requirements (FIS)<br><br>Items of FAIM – chance of accidental ingestion,	FIS/non-FIS caregivers of children with FA (clinician diagnosed)	Scores on FIS, FAIM + history of food-induced anaphylaxis (FIA)	Caregivers classified as FIS reported increased perceived risk (p=0.001) of accidental ingestion (41% versus 17%), severe reaction (45% versus 24%), and death (38% versus 12%).<br><br>FIS status was associated with child FIA (v2 = 5.54,	Findings may reflect experiences specific to FA, such as the economic burden of obtaining FA-safe foods; access to FA-safe foods; and FA related anxiety.<br><br>Future research is needed to examine FA risks and the

		+ Food Allergy Independent Measure (FAIM)		adverse reaction, dying, and managing reaction well.			P = 0.02, phi = 0.19; 44% versus 24%).<br><br>FIS associated (p<0.01) with child history of FIA (phi = 0.19) utilization of food assistance programs (52% versus 8%) and food banks (74% versus 28%).	impact of FIS on nutritional status.<br><br>Limitations all data collected online self-report and cross sectional. Longitudinal needed.<br>FA not physician-confirmed
Van der Velde, 2012, The Netherlands	Longitudinal, psychometric measurement (1 month before and 6 months after FC) Case/control<br><br>All participants referred by specialist	Food Allergy Quality of Life Questionnaire–Adult Form (FAQLQ-AF), the Food Allergy Quality of Life Questionnaire–Teenager Form (FAQLQ-TF), and the Food Allergy Quality of Life Questionnaire – Child Form (FAQLQ-CF)		Allergen avoidance ; Risk of accidental exposure (RAE);Emotional impact (EI);Dietary restrictions	N=221	To assess the longitudinal validity and responsiveness of the FAQLQ-AF, FAQLQ-TF, and FAQLQ-CF and to assess the impact of a DBPCFC on HRQL.	Sensitivity and responsiveness demonstrated.<br>HRQL scores improved after a DBPCFC, with greater improvements in HRQL scores after negative OFC outcome than a positive OFC outcome.<br>Significant correlations were shown between the change (follow-up minus baseline) in FAQLQ and FAIM scores supporting longitudinal validity (r= 0.35-0.71, p<0.05)	Of interest, even a positive challenge improved QoL in children and adults. It is possible that their recognition of what would happen in the event of an unintentional ingestion removed the uncertainty with which they had lived on a daily basis. The same was not seen in teenagers with a positive challenge, suggesting that their QoL concerns lay more with the fact that they have food allergy than what would happen with ingestion. Patients with an uncertain history or positive test result in the absence of a positive history should be considered for food challenge in a controlled setting by those experienced with the procedure.
Warren, 2015, US	Cross sectional<br><br>Eligible families were those	Family Empowerment and FAQOL Parental Burden scales	Food allergy was defined by stringent criteria,	Total Scores on FAQLQ-PB and Parental Empowerment	The 876 children included in this study	The differences in food allergy-related quality of life (FAQOL) and empowerment of	Mothers reported greater empowerment (P < .001) and lower FAQOL (P < .001) compared with	Parental empowerment and FAQOL vary significantly among mothers and fathers of children

	having 1 or 2 parents with at least 1 biological child (21 years old) with food allergy who were willing to participate. Families were recruited through general medical and allergy specialty clinics, community support groups, and media advertisements.		including reaction history, skin prick testing, and specific IgE.		were enrolled as part of the Chicago Family Cohort food allergy study.	mothers and fathers of a large cohort of children with food allergy according to allergen severity, type, or comorbidities.	fathers, regardless of allergen severity, type, or comorbidities. However, parental empowerment was not significantly associated with FAQOL for mothers or fathers. Although parents of children with peanut, cow milk, egg, and tree nut allergies were similarly empowered, milk and egg allergies were associated with lower FAQOL (P < .01). Parental concern in the QOL assessment was greatest for items involving fear of allergen exposure outside the home	with food allergy. Greater effects on FAQOL were seen for milk and egg compared with other food allergies. Although parents of children with food allergy might be empowered to care for their child, they continue to experience impaired FAQOL owing to fears of allergen exposure beyond their control. Given the ubiquity of cow's milk and egg in the Western diet and frequent confusion between true IgE-mediated allergy and food intolerance, the avoidance of these allergens poses a greater challenge to parents and is associated with decreased parental FAQOL. The sample was comprised of predominantly white, upper-income married couples and, as such, might not be generalizable to the US population. Future research needed to evaluate FA in more ethnically and socioeconomically diverse groups.

List of abbreviations: AA=Allergen avoidance; DBPCFC= double-blind placebo-controlled food challenge; EI= Emotional impact; FA= food allergy; FAQLQ-AF= Food allergy quality of life questionnaires adult form; FAQLQ-CF= Food allergy quality of life questionnaires child form; FAQLQ-PF= Food allergy quality of life questionnaires parent proxy form; FAQLQ-TF= Food allergy quality of life questionnaires teenager form; FAIM= food allergy independent measure; FIA= food-induced anaphylaxis; FIS= food insecurity; HRQL= health-related quality of life; MID= minimally

important difference; NR= not reported; IFQ= Impact on Family Questionnaire; OAS=oral allergic syndrome; OFC= oral food challenge; OR= odds ratio; QoL=quality of life; RAE= Risk of accidental exposure; RR= relative risk; SDL=Social & Dietary Limitations; SNAP= Supplemental Nutrition Assistance Program; VAS= visual analogue scale.

Table S5. Detailed characteristics of economic evaluation studies (n=2).
(A) Methods of costing studies

Author, Year (country)	Studies that report different grades of food allergy severity (mild, moderate, and severe), and not general food allergy	Study Design	Year(s) of data collection	Data sources	Setting	Sample size	Perspective	Categories of cost	Cost measurement methodology	Sources of cost	Currency, price date	Methods of sensitivity analyses
Flabbee et al., 2008 (France)	Study based on "402 patients of severe anaphylaxis cases documented by the Allergy Vigilance Network."	Cost study	January 2004 to end of June 2006	Allergy Vigilance Network PMSI Program database (recordings of hospital admissions)	Hospital and general Practice	402 cases (181 cases in 2004; 108 cases in 2005; 113 cases in 2006)	National costs	• Direct medical costs: consultations, use of emergency units, examinations, hospitalizations, and drugs. nonmedical costs: transport, special diets, etc.<br>• Indirect costs: absenteeism (3 days)	Each patients report was analysed, and then extrapolated to national annual costs using reported hospital anaphylaxis codes: *"T780: anaphylactic shock because of adverse food reaction, T782: anaphylactic shock, not specified, T805: anaphylactic shock because of serum/vaccine/immunization, T 886: anaphylactic shock because of adverse drug reactions, T882: anesthetic shock".*	Direct costs: public hospital fees and the current fees of General practitioner emergency visits. Indirect costs: calculated on basis of Belgian costs, statbel.gov.be	Euro, currency year not indicated.	N/A

Fox et al., 2013 (Europe: Greece, Iceland, Poland, Spain, Czech Republic, France, Italy, The Netherlands, and UK)	Those with food-specific immunoglobulin E were defined as having probable allergy.<br><br>"There is evidence that the cost of health services for those with moderate food allergy (category 3) is likely to be 68% higher than for those with the mildest symptoms of food allergy. The cost of health services for those with severe food allergy (category 4) is predicted to be twice that of those with mild	Age-specific Case-control study. Cases were respondents with possible food allergy (from stage 2). Objectives: Compare healthcare costs of people with food allergy compared to others with no adverse reactions to food. Assess if levels of severity (using Mueller clinical severity grading scale: 4 category scale -	January 2007 until July 2009	EuroPrevall centres, use of FA-ECOQ. Economic data: collected from 4 EuroPrevall centres in Greece, Iceland, Poland and Spain, for cases with possible food allergy and controls. Data from cases with probable food allergy were obtained from 7 EuroPrevall centres in,	General Practitioners' patient lists, city council registration databases, local authority/hospital debases, and primary schools.	In stage 2 of the study, 1411 participants, 674 Adults (aged 20–54 years) and 737 parents that represented their children (aged 7–11 years) participated.<br>In stage 3 of the study (cases only) 271 participants completed the FA-ECOQ.	Health care commissioners.	Direct costs: number of primary care visits, outpatient visits, hospital inpatient stays (number of days admitted), use of ambulance services, and prescribed medications.	Respondents to the FA-ECOQ provided information reading healthcare use, which was then multiplied by relevant unit costs.	WHO-CHOICE database for costs of reported health services during the past year by participants.	2016, International dollar I$ (Geary–Khamis dollar)	N/A

	food allergy."	Grades 1 to 4) had an impact on costs.		Poland, Spain, Czech Republic, France, Italy, The Netherlands and UK.								

Abbreviations: CAST= the Cellular Allergen Stimulation Test; EUROPREVALL= The prevalence, cost and basis of food allergy across Europe; FA-ECOQ=the Food Allergy Economic Questionnaire; FRGB001 Allergic test by intradermal injection of several substances administered in increasing concentration with at least 2 dilutions; FRGB002= Allergic test by intradermal injection of a substance administered in increasing concentration; FRGB003= Allergic test by epidermal bite with substances administered at fixed concentration; FRGB004= Allergic skin prick test with native foods; FRGB005= Allergic test by intradermal injection of substances administered at fixed concentration; Fx5= Multitest Fx5 in Food Allergy; WHO-CHOICE= the World Health Organization CHOosing Interventions that are Cost-Effective.

(B): Results of costing studies

Author, Year (country)	Economic costs	Resource consequences	Sensitivity analyses

<table>
<tr>
<td>Flabbee et al., 2008 (France)</td>
<td>Costs of medical procedures for management of anaphylaxis:
<table>
<tr><td>General Practitioner</td><td></td></tr>
<tr><td>Mean cost of emergency visit</td><td>95.90€</td></tr>
<tr><td>Hospitalization</td><td></td></tr>
<tr><td>Emergency ambulance brigade called (without later hospitalisation)</td><td>200.00€</td></tr>
<tr><td>Visit to emergency unit (<5 h)</td><td>462.00€</td></tr>
<tr><td>Visit to emergency unit and hospitalization exceeding 5 h</td><td>1140.00€/day</td></tr>
<tr><td>Emergency ambulance brigade and emergency unit = equivalent costs
Hospitalization in medical unit
Hospitalization in resuscitation or intensive care unit</td><td>

779.00€/day
2115.00€/day</td></tr>
<tr><td>Allergy screening</td><td></td></tr>
<tr><td>Allergy tests</td><td></td></tr>
<tr><td>Prick-tests (FGRB003 or FGRB004)</td><td>28.80€ or 31.51€</td></tr>
<tr><td>IDR FGRB001, FGRB002 or FGRB005</td><td>30.49€ or 34.16€</td></tr>
<tr><td>Laboratory tests</td><td></td></tr>
<tr><td>Specfic IgE analysis</td><td>14.85€</td></tr>
<tr><td>Serum tryptase assay</td><td>27.00€</td></tr>
<tr><td>Serum histamine</td><td>40.50€</td></tr>
<tr><td>Fx5</td><td>14.85€</td></tr>
<tr><td>CAST</td><td>56€</td></tr>
<tr><td>Basophil activation test</td><td>40€</td></tr>
<tr><td>Leukocyte histamine release test</td><td>56.70€</td></tr>
<tr><td>Hospitalization</td><td></td></tr>
<tr><td>For challenge tests</td><td>779.00€/day</td></tr>
<tr><td>Emergency kits</td><td></td></tr>
<tr><td>Adult (Anapen + Ventoline + Solupred oro + Aerius)</td><td>96.95€</td></tr>
<tr><td>Child (Anapen + Ventoline + Celestene+ Aerius)</td><td>58.79€</td></tr>
</table>
Direct cost of severe anaphylaxis ranged from 74.88€ to 4445.47€. The mean direct cost was 1580€/patient, and the mean indirect cost was 315€. The total cost was 1895€. Extrapolated annual national cost was 4,789,500€.</td>
<td>Hospital statistics for 2003–2005 according to anaphylaxis coding:
<table>
<tr><td>ICD 10 code</td><td>Classification</td><td>2003</td><td>2004</td><td>2005</td></tr>
<tr><td>T780</td><td>Anaphylactic shock due to adverse food reaction</td><td>153</td><td>212</td><td>236</td></tr>
<tr><td>T782</td><td>Anaphylactic shock, not specified</td><td>1426</td><td>1708</td><td>1703</td></tr>
<tr><td>T805</td><td>Anaphylactic shock due to serum/vaccine/immunization</td><td>16</td><td>15</td><td>11</td></tr>
<tr><td>T886</td><td>Anaphylactic shock due to adverse drug reactions</td><td>46</td><td>553</td><td>535</td></tr>
<tr><td>T882</td><td>Anesthetic shock</td><td>69</td><td>78</td><td>90</td></tr>
</table></td>
<td>N/A</td>
</tr>
</table>

Fox et al., 2013 (Europe: Greece, Iceland, Poland, Spain, Czech Republic, France, Italy, The Netherlands, and UK)	The average healthcare costs for adults with possible food allergy was I$2016 compared with I$1089 for controls, a difference of I$927.<br>For children the average cost for cases was I$2197 and for controls it was I$863, a difference of I$1334.<br>Average yearly cost of health care for 766 cases of possible and probable food allergy in the nine participating centres was I$1778.<br><br>The cost of healthcare services for people with moderate food allergy (category 3) was estimated to be 68% higher than for those with mild symptoms.<br>The costs for those with severe food allergy (category 4) was predicted to be twice the amount for people with mild food allergy.	Adults:<br>Cases with possible food allergy visited health professionals, on average, 11.17 (SD = 16.14) times per year.<br>Controls visited health professionals on average 7.11(SD = 12.80) per year<br>Children:<br>Cases visited health professionals 10.75 times per year (SD = 13.23)<br>Controls visited health professionals 6.56 (SD = 9.78) times per year.	

Abbreviations: CAST= the Cellular Allergen Stimulation Test; EUROPREVALL= The prevalence, cost and basis of food allergy across Europe; FA-ECOQ=the Food Allergy Economic Questionnaire; FRGB001 Allergic test by intradermal injection of several substances administered in increasing concentration with at least 2 dilutions; FRGB002= Allergic test by intradermal injection of a substance administered in increasing concentration; FRGB003= Allergic test by epidermal bite with substances administered at fixed concentration; FRGB004= Allergic skin prick test with native foods; FRGB005= Allergic test by intradermal injection of substances administered at fixed concentration; Fx5= Multitest Fx5 in Food Allergy; WHO-CHOICE= the World Health Organization CHOosing Interventions that are Cost-Effective.

Table S6. Critical appraisal of included studies.

(A) Critical appraisal of included primary studies assessing symptom-specific severity of food allergy (n=23) assessed by the Effective Public Health Practice Project (EPHPP)

	Design	Selection Bias			Study Design			Confounders			Blinding			Data Collection Method			Withdrawals and Dropouts			Global Rating		
		S	M	W	S	M	W	S	M	W	S	M	W	S	M	W	S	M	W	S	M	W
Amin, 2012, USA	Cohort	✓				✓		✓				NA		✓				NA		✓		
Astier, 2006, France & USA	Case-control			✓		✓				✓		NA		✓				NA				✓
Bernard, 2003, France, USA	Cohort			✓		✓		✓				NA		✓				NA			✓	
Boyano-Martìnez, 2009, Spain	Cross-sectional	✓					✓	✓				NA		✓				NA			✓	
Boyano-Martìnez, 2012, Spain	Cross-sectional	✓					✓	✓				NA		✓				NA			✓	
Braganza, 2006, Australia	Case-series			✓	✓			✓				NA		✓				NA			✓	
Brown, 2004, Australia	Case-series		✓				✓	✓				NA		✓				NA			✓	
Brown, 2013, Australia	Case-series			✓			✓	✓				NA		✓				NA				✓
Clark, 2004, USA	Cohort	✓				✓		✓				NA		✓				NA		✓		
Corriger, 2019, France	Case-series		✓				✓			✓		NA		✓				NA				✓
Ewan, 2001, UK	Cohort	✓				✓		✓				NA		✓			✓			✓		
Hourihane, 1997, UK	Cross-sectional	✓					✓			✓		NA		✓				NA				✓
Hourihane, 2005, UK	Cross-sectional	✓					✓	✓			✓			✓				NA			✓	
Itazawa, 2020, Japan	Case-series			✓			✓	✓				NA		✓				NA				✓
Macdougall, 2002, UK	Cohort		✓			✓				✓		NA		✓				NA			✓	
Moro-Moro, 2011, Spain	Cohort		✓			✓		✓				NA			✓			NA		✓		

Primeau, 2000, Canada	Case-control		✓			✓		✓				NA		✓				✓			✓	
Tejedor-Alonso, 2013, Spain	Cohort	✓				✓		✓				NA		✓				✓		✓		
Van Erp, 2013, The Netherlands	Cohort	✓				✓		✓			✓			✓				NA		✓		
Van Erp, 2014, The Netherlands	Cohort	✓				✓			✓		✓			✓				NA		✓		
Vetander, 2014, Sweden	Cohort		✓			✓		✓				NA			✓			NA		✓		
Virkud, 2019, USA	Cohort	✓				✓		✓				NA			✓			NA		✓		
Ye, 2015, Korea	Cohort	✓				✓		✓				NA		✓				NA		✓		

Abbreviations:

EPHPP	the Effective Public Health Practice Project
S	Strong
M	Moderate
W	Weak
NA	Not applicable
NR	Not reported

(B) Critical appraisal of included primary studies assessing Food Allergy related-Quality of life measures (n=7) assessed by the Effective Public Health Practice Project (EPHPP).

Study, country	Design	Selection Bias			Study Design			Confounders			Blinding			Data Collection Method			Withdrawals and Dropouts			Global Rating		
		S	M	W	S	M	W	S	M	W	S	M	W	S	M	W	S	M	W	S	M	W
Dantzer 2018, USA	Cross-sectional			✓			✓		✓			NA		✓				NA				✓
DunnGalvin, 2010, International	Cross-sectional	✓					✓	✓				NA		✓			✓				✓	
Flokstra-de Blok, 2010, International	Cross-sectional	✓					✓	✓				NA		✓			✓				✓	
Goossens, 2011, International	Cross-sectional			✓			✓		✓			NA		✓			✓					✓

Tackett, 2018, USA	Cross-sectional			✓			✓		✓		**NA**	✓				✓				✓
van der Velde, 2012 The Netherlands	Cross-sectional	✓					✓	✓			NA	✓			✓				✓	
Warren, 2015, USA	Cross-sectional	✓					✓	✓			NA	✓			✓				✓	

Abbreviations:

EPHPP	the Effective Public Health Practice Project
S	Strong
M	Moderate
W	Weak
NA	Not applicable
NR	Not reported

Table S7. Predictors described in included primary studies assessing symptom-specific severity of food allergy

Study	Predictors for severe food-induced allergic reactions											Details
	Host-related				Food allergen -related							
(First author, year of publication, country)	gender	older age	asthma	drugs	recurrence of reaction	severity of previous reaction	type of food	total IgE	sIgE	SPT	polisensitization (same source)	
Amin, 2012, USA												• type of food:<br>The highest risk of severe ARs as initial AR for:<br>✓ peanuts (OR=1.76, 95%CI: 0.9–3.45)<br>✓ shellfish (OR= 1.54, 95%CI: 0.49–5.64)<br>The lowest for: sesame, soy, and wheat
Astier, 2006, France & USA												• sIgE (rAra h1, rAra h2, rAra h3): NS<br>• SPT (rAra h1, rAra h2, rAra h3): NS<br>• Pts monosensitized to rAra h 2 had a significantly lower severity score than polysensitized pts (i.e. rAra h 2 and rAra h 1 and/or rAra h 3)(P < .02)
Bernard, 2003, France, USA												• sIgE (to whole peanut proteins, rAra h1, rAra h2): were significantly higher in moderate or severe AR group vs mild AR one
Boyano-Martìnez, 2009, Spain$^{\alpha}$												• sIgE to cow's milk: significantly higher in children with severe ARs than in those with moderate/ mild/ no ARs<br>• sIgE to casein: similar association<br>• asthma: The frequency of severe ARs compared with moderate, mild, or no ARs was 10-fold higher in asthmatic children (OR, 10.19; 95% CI, 1.13-91.54; P .022).
Boyano-Martìnez, 2012, Spain$^{\beta}$												• gender: NS<br>• severity of the first AR with egg: NS<br>• asthma: NS<br>• total IgE were significantly lower in pts with moderate/severe ARs (adjusted odds ratio for every 1-unit increase in the decimal logarithm, 0.16; 95% CI, 0.05-0.54; P=.001)<br>• sIgE to egg white higher in children with moderate or severe ARs (adjusted odds ratio for every 0.1-unit increase in the decimal logarithm, 1.15; 95% CI, 1.03-1.28; P=.008)

Brown, 2004, Australia												• age: NS<br>• ACE inhibitor: NS<br>• β-blocker: NS<br>• medication: NS
Ewan, 2001, UK												• age: pts with severe AR (grade 4 and 5) during follow-up were older (median 18 years *vs* 9 years; p<0.05). The 3 pts with grade 5 AR were aged 27-41 yrs. Similarly, considering the first AR, the median age of onset was 2 yrs for mild ARs and 11 yrs for severe ARs (grade 4-5, p<.005).
Hourihane, 1997, UK												• Age: mild symptoms were more common in children and severe symptoms more common in adults (Kruskal-Wallis one-way ANOVA, P = 0.0002)<br>• Asthma: Pts who reported a Hx of asthma were more likely to suffer severe ARs (x2=17.9, P.00013). Wheeze was the most common severe $, (~ 40% of pts on both first and last ARs).<br>• SPT: NS<br>• sIgE to peanut: NS
Hourihane, 2005, UK												• sIgE to peanut and challenge score correlated significantly in the whole group but stronger in adults than in children, despite the median values of peanut sIgE being similar. In adults Spearman's r-value increased to 0.766 (P=0.001, compared with children (r=0.49, P=.018).<br>• asthma: NS<br>• age: NS<br>• gender: NS
Macdougall, 2002, UK												• asthma: p=0.0002<br>• severity of previous AR: NS
Van Erp, 2013, The Netherlands												• age: NS<br>• gender: NS<br>• asthma: NS<br>• sIgE: NS<br>• previous AR to peanut: NS
Virkud, 2019, USA		*							*	*		• sIgE, SPT and age at challenge combined demonstrated good predictive value for grade 2/3 allergic reactions by AUC (0.83)
Ye, 2015, Korea												• Type of food:<br>✓ Wheat can be the only predictor of severe anaphylaxis (OR 2.425, 95% CI 1.054-5.581, p<0.037)<br>✓ Seafood: NS<br>✓ Vegetable: NS<br>✓ Meat: NS

List of abbreviations: AR, food-induced allergic reaction; AUC, area under curve; CI, confidential interval; DBPCFC, double-blind placebo-controlled food challenge; NR, not reported; NS,non-significant association; OR, odds ratio; RR, relative risk; sIgE, specific immunoglobulin E; SPT, skin prick test; $, symptom(s)

List of included primary studies not reporting any predictor for severe food-induced allergic reaction: Braganza, 2006; Brown, 2013‡; Clark 2004; Corriger, 2019‡; Furlong 2001; Itazawa 2020; Moro-Moro, 2011; Primeau, 2000, Sicherer, 1999; Tejedor-Alonso, 2013; Van Erp, 2014; Vetander, 2014; Warren, 2019.

‡ data reported cumulatively for different triggers

* combination of predictors

[α] A strong association was found between asthma and high sIgE levels to milk and casein. Due to the low sample size it is unclear if asthma is an independent factor for having severe ARs or is just a modifier of the effect of sIgE levels.

[β] These results were similar when the risk of suffering moderate or severe ARs was compared with that of suffering mild or no ARs.

COLOUR LEGEND

- (red) significant risk factor for severe food-induced allergic reaction
- (yellow) non-significant risk factor for severe food-induced allergic reaction
- (green) protective factor for severe food-induced allergic reaction
- (white) analysis not reported

Table S8: Direct medical costs for severe anaphylaxis management (cost 2020 €) reported in Flabbee et al., 2008[66]

	Medical costs: Cost 2020 €
General Practitioner	
Mean cost of emergency visit	€114.64
Hospitalization	
Emergency ambulance brigade called (without later hospitalisation)	€239.08
Visit to emergency unit (<5h)	€552.27
Visit to emergency unit and hospitalization exceeding 5h	€1,362.75/day
Emergency ambulance brigade and emergency unit: Hospitalization in medical unit	€931.21/day
Emergency ambulance brigade and emergency unit: Hospitalization in resuscitation or intensive care unit	€2,528.25/day
Allergy screening	
Allergy tests	
Prick-tests (FGRB003 or FGRB004)	€34.43 or €37.67
IDR FGRB001, FGRB002 or FGRB005	€36.45 or €40.83
Laboratory tests	
Specfic IgE analysis	€17.75
Serum tryptase assay	€32.28
Serum histamine	€48.41
fx5	€17.75
CAST	€66.94
Basophil activation test	€47.82
Leukocyte histamine release test	€67.78
Hospitalization	
For challenge tests	€931.21/day

Emergency kits	
Adult (Anapen® $_{0,3}$ + Ventoline® + Solupred® oro + Aerius®)	€115.89
Child (Anapen® $_{0,15}$ + Ventoline® + Celestene® + Aerius®)	€70.28
